# Longitudinal Analysis of the Utility of Liver Biochemistry as Prognostic Markers in Hospitalized Patients With Corona Virus Disease 2019

**DOI:** 10.1002/hep4.1739

**Published:** 2021-07-10

**Authors:** Tingyan Wang, David A. Smith, Cori Campbell, Steve Harris, Hizni Salih, Kinga A. Várnai, Kerrie Woods, Theresa Noble, Oliver Freeman, Zuzana Moysova, Thomas Marjot, Gwilym J. Webb, Jim Davies, Eleanor Barnes, Philippa C. Matthews

**Affiliations:** ^1^ National Institute for Health Research (NIHR) Oxford Biomedical Research Centre Oxford United Kingdom; ^2^ Nuffield Department of Medicine University of Oxford Oxford United Kingdom; ^3^ Oxford University Hospitals National Health Service Foundation Trust Oxford United Kingdom; ^4^ Department of Computer Science University of Oxford Oxford United Kingdom; ^5^ Oxford Liver Unit, Translational Gastroenterology Unit John Radcliffe Hospital Oxford University Hospitals Oxford United Kingdom; ^6^ Cambridge Liver Unit Addenbrooke's Hospital Cambridge United Kingdom; ^7^ Department of Infectious Diseases and Microbiology Oxford University Hospitals National Health Service Foundation Trust Oxford United Kingdom

## Abstract

The association of liver biochemistry with clinical outcomes of severe acute respiratory syndrome coronavirus 2 (SARS‐CoV‐2) infection is currently unclear, and the utility of longitudinally measured liver biochemistry as prognostic markers for mortality is unknown. We aimed to determine whether abnormal liver biochemistry, assessed at baseline and at repeat measures over time, was associated with death in hospitalized patients with COVID‐19 compared to those without COVID‐19, in a United Kingdom population. We extracted routinely collected clinical data from a large teaching hospital in the United Kingdom, matching 585 hospitalized patients who were SARS‐CoV‐2 real‐time reverse transcription‐polymerase chain reaction (RT‐PCR) positive to 1,165 hospitalized patients who were RT‐PCR negative for age, sex, ethnicity, and preexisting comorbidities. A total of 26.8% (157/585) of patients with COVID‐19 died compared to 11.9% (139/1,165) in the group without COVID‐19 (*P* < 0.001). At presentation, a significantly higher proportion of the group with COVID‐19 had elevated alanine aminotransferase (20.7% vs. 14.6%, *P* = 0.004) and hypoalbuminemia (58.7% vs. 35.0%, *P* < 0.001) compared to the group without COVID‐19. Within the group with COVID‐19, those with hypoalbuminemia at presentation had 1.83‐fold increased hazards of death compared to those with normal albumin (adjusted hazard ratio [HR], 1.83; 95% confidence interval [CI], 1.25‐2.67), while the hazard of death was ~4‐fold higher in those aged ≥75 years (adjusted HR, 3.96; 95% CI, 2.59‐6.04) and ~3‐fold higher in those with preexisting liver disease (adjusted HR, 3.37; 95% CI, 1.58‐7.16). In the group with COVID‐19, alkaline phosphatase (ALP) increased (R = 0.192, *P* < 0.0001) and albumin declined (R = −0.123, *P* = 0.0004) over time in patients who died. *Conclusion:* In this United Kingdom population, liver biochemistry is commonly deranged in patients with COVID‐19. Baseline hypoalbuminemia and rising ALP over time could be prognostic markers for death, but investigation of larger cohorts is required to develop a better understanding of the relationship between liver biochemistry and disease outcome.

**Abbreviations**
ACE2: angiotensin‐converting enzyme 2
ALP: alkaline phosphatase
ALT: alanine aminotransferase
AST: aspartate aminotransferase
AUC: area under the curve
BMI: body mass index
BR: bilirubin
CHD: coronary heart disease
CI: confidence interval
CKD: chronic kidney disease
COVID‐19: corona virus disease 2019
CRP: C‐reactive protein
DM: diabetes mellitus
GGT: gamma‐glutamyltransferase
HIC: Health Informatics Collaborative
HR: hazard ratio
HTN: hypertension
ICU: intensive care unit
INR: international normalized ratio
IQR: interquartile range
K‐M: Kaplan‐Meier
LLN: lower limit of normal
NIHR: National Institute for Health Research
OUH: Oxford University Hospitals
ROC: receiver operating characteristic
RT‐PCR: reverse transcription‐polymerase chain reaction
SARS‐CoV‐2: severe acute respiratory syndrome coronavirus 2
ULN: upper limit of normal
WHO: World Health Organization

There were over 100 million confirmed cases of severe acute respiratory syndrome coronavirus 2 (SARS‐CoV‐2) infection within a year of the first case being reported, and ~2.5 million deaths had been reported globally by the end of February 2021. The United Kingdom was one of the worst affected countries, with over 4 million confirmed cases and over 121,000 deaths reported in this time period.^(^ ^1^ ^)^ The clinical syndrome caused by SARS‐CoV‐2, corona virus disease 2019 (COVID‐19), primarily affects the respiratory system, but other organs, including the heart, gastrointestinal tract, and liver, may also be affected, and a systemic sepsis syndrome may develop.^(^ ^2^ ^)^

Data on liver biochemistry in patients with COVID‐19 have been reported from China, the United States, and Italy. These studies report that 37%‐69% of patients with COVID‐19 had at least one abnormal liver biochemistry on hospital admission^(^ ^3^, ^4^, ^5^, ^6^, ^7^, ^8^, ^9^ ^)^ while 93% had at least one abnormal liver biochemistry over the course of disease.^(^ ^6^ ^)^ In particular, the prevalence estimates of elevated alanine aminotransferase (ALT), aspartate aminotransferase (AST), and bilirubin (BR) in patients hospitalized with COVID‐19 are 9%‐28%, 14%‐35%, and 6%‐23%, respectively.^(^ ^3^, ^4^, ^5^, ^6^, ^10^, ^11^, ^12^ ^)^ Some studies have reported that liver biochemistry abnormalities are associated with longer hospital stay^(^ ^4^ ^)^ or clinical severity,^(^ ^3^, ^12^, ^13^ ^)^ whereas other studies have not found a relationship between liver biochemistry and severity.^(^ ^10^ ^)^ The set of liver biochemistry tests reported for patients with COVID‐19 varies; ALT, AST, and total BR are typically included, with alkaline phosphatase (ALP) and gamma‐glutamyltransferase (GGT) less frequently reported.

Albumin is a nonspecific marker of liver function and has been less consistently assessed; it is typically reported in patient baseline characteristics, with limited investigation of its utility as a prognostic marker. However, a recent meta‐analysis of 20 retrospective cohort studies from China reported lower baseline albumin levels in patients with severe COVID‐19 compared to mild cases but with significant heterogeneity between studies.^(^ ^14^ ^)^ Another meta‐analysis demonstrated that hypoalbuminemia could be included in prognostic machine‐learning models to predict severe COVID‐19 or mortality.^(^ ^15^ ^)^

Several studies have investigated potential associations between liver biochemistry and death in patients with COVID‐19^(^ ^6^, ^8^, ^9^, ^10^, ^11^, ^16^, ^17^ ^)^ or included liver biochemistry in the development of predictive models.^(^ ^18^, ^19^, ^20^, ^21^ ^)^ A report from Italy showed that ALP >150 U/L at hospital admission (without adjusting for relevant confounders) was associated with clinical deterioration in 292 patients with COVID‐19,^(^ ^10^ ^)^ and another study from the United States reported that peak ALT >5 times upper limit of normal (ULN) during admission was associated with death in a cohort of 2,273 patients with COVID‐19.^(^ ^11^ ^)^ However, another U.S. study reported that elevations in ALT and AST on admission were associated with length of stay, intensive care unit (ICU) admission, and intubation, but not death.^(^ ^6^ ^)^ Studies from Wuhan, China, did not find associations of ALT^(^ ^18^, ^19^ ^)^ or AST^(^ ^19^ ^)^ elevation on admission with death in patients with COVID‐19, while several studies have reported associations of elevated total BR^(^ ^16^, ^19^ ^)^ and low albumin on admission^(^ ^19^, ^20^, ^21^ ^)^ with risk of death. Other studies considered abnormality of liver tests as one composite variable^(^ ^7^ ^)^ or a composite endpoint (ICU admission or death) as the primary outcome^(^ ^8^, ^9^, ^17^ ^)^ without specifying which individual liver test abnormalities were associated with risk of death. Given these variable associations between liver biochemistry and COVID‐19 outcomes, the prognostic value of liver biochemistry derangement in COVID‐19 needs further evaluation.

Having established a clinical data pipeline through the National Institute for Health Research (NIHR) Health Informatics Collaborative (HIC),^(^ ^22^, ^23^ ^)^ our tertiary referral hospital in the United Kingdom is strongly placed to undertake analyses using electronic health data from hospitalized patients. Using this resource, we aimed to determine the prevalence of deranged liver biochemistry at baseline and over the disease course in patients with COVID‐19 with comparison to a matched group of patients without COVID‐19 admitted during the same period. We also aimed to determine whether baseline liver biochemistry derangement was associated with risk of death in patients with COVID‐19 and to compare longitudinal changes in liver biochemistry between patients with COVID‐19 who died and those who survived.

## Participants and Methods

### Data Collection

We used routinely collected clinical data from Oxford University Hospitals (OUH) National Health Service (NHS) Foundation Trust, a large teaching hospital trust in the southeast of the United Kingdom that has ~1,000 in‐patient beds. The data are collected by the local NIHR HIC team in Oxford, being drawn automatically from operational systems into a data warehouse and linked to produce a comprehensive record for each patient with a data validation process, as described in our HIC methods paper.^(^ ^23^ ^)^ The management of the data set is governed by the NIHR HIC Data Sharing Framework. All the data used for this study were provided in anonymized form by OUH NHS Foundation Trust with the prior approval of the Trust Information Governance Team following the satisfactory completion of a data protection impact assessment.

The data extracted for this study included detailed information on demographics, body mass index (BMI), emergency admissions, blood test results, diagnostic codes, procedures, ICU admission, prescriptions, medicines administration, and discharge destination/outcome for all patients admitted to OUH between January 1, 2020, and August 21, 2020.

### Inclusion and Exclusion Criteria

To select eligible data for adults with/without COVID‐19, the inclusion criteria were as follows: (a) at least one real‐time reverse transcription‐polymerase chain reaction (RT‐PCR) nose/throat swab having been undertaken, which is the validated clinical test being deployed through our hospital diagnostic microbiology laboratory (patients with COVID‐19 were defined by at least one positive result; patients negative for COVID‐19 were defined by the absence of a positive result); (b) age ≥18 years when tested; (c) hospitalized patients; and (d) at least one episode of liver biochemistry recorded at the time or after the RT‐PCR test (the set of liver biochemistry routinely tested by OUH clinical biochemistry laboratory comprises ALT, ALP, albumin, and BR; AST and GGT are not routinely tested). Exclusion criteria were as follows: (a) SARS‐CoV‐2 RT‐PCR test results reported as invalid; (b) missing age; or (c) pregnancy (antenatal/delivery/postpartum). Patients were followed until death or until the last available clinical record.

### Definitions

We defined baseline as the date of the first positive SARS‐CoV‐2 RT‐PCR test for a patient with COVID‐19 and the date of the first negative test for a patient without COVID‐19.

The normal ranges set by the hospital biochemistry laboratory are as follows: ALT, 10‐45 IU/L; ALP, 30‐130 IU/L; BR, 0‐21 µmol/L; albumin, 32‐50 g/L. The reference ranges for other blood tests are provided in Supporting Table S1. We defined baseline liver biochemistry (ALT, ALP, BR, or albumin) as the liver biochemistry measured within 7 days of a SARS‐CoV‐2 RT‐PCR test and baseline derangement as at least one abnormal result at this time point. We defined peak/nadir liver biochemistry derangement as abnormality of the highest/lowest value recorded, respectively, at any point during follow‐up. We defined liver biochemistry recovery as normalization following derangement. The primary outcome was death during follow‐up, and secondary outcomes included ICU admission and invasive ventilation.

Preexisting comorbidities were defined by a historic diagnosis of a disease before baseline; diagnosis codes retrieved are provided in Supporting Table S2. A full list of prescribed drugs searched for data extraction is provided in Supporting Table S3.

### Statistical Analysis

Statistical analysis was performed using R version 4.0.2. All significance tests performed were two‐sided. *P* < 0.05 was deemed statistically significant.

#### Propensity Score Matching

We identified eligible adults and conducted a propensity score matching process to ensure the group with COVID‐19 was comparable to the group without COVID‐19 in terms of demographics and preexisting conditions. We used the following variables to calculate propensity scores: age, sex, ethnicity, and preexisting comorbidities (liver disease, diabetes mellitus [DM], hypertension [HTN], coronary heart disease [CHD], chronic kidney disease [CKD], and cancer). We performed propensity score matching using the package MatchIt, with the nearest neighbor method applied, the matching ratio and caliper size set as 1:2 and 0.1, respectively, and without replacement.

#### Comparison of Patients With and Matched Patients Without COVID‐19

For continuous variables, we calculated median and interquartile range (IQR) or mean and SD and used the Wilcoxon test or *t* test for comparison. For categorical variables, we computed number and percentage and used the chi‐square or Fisher’s exact test for comparison. We used the Shapiro‐Wilk test and graphical methods for normality checks. We further stratified patients with COVID‐19 by the severity of disease into mild/moderate and severe/critical subgroups (using respiratory rate and oxygen saturation thresholds of ≤30 breaths/minute and ≥90%, respectively, for mild/moderate illness; and thresholds of >30 breaths/minute or <90%, respectively, for severe illness, as established by the World Health Organization [WHO]).^(^ ^24^ ^)^ We then compared liver biochemistries and outcomes of each severity subgroup with COVID‐19 to those of the group without COVID‐19.

#### Investigation on Whether Liver Biochemistry Predicts Outcomes in Patients With COVID‐19

We compared the presence of clinical outcomes in the groups with and without COVID‐19. We also performed Kaplan‐Meier (K‐M) analysis to compare the survival probability over time between the two groups. Within the groups with and without COVID‐19, we compared demographics, BMI, comorbidities, and baseline and peak/nadir liver biochemistry between those who died versus those who survived. We then performed K‐M analysis to compare the survival probabilities over time between subgroups with and without deranged baseline liver biochemistry. We used univariate and multivariate Cox proportional hazards models to investigate whether liver biochemistry predicted death, reporting hazard ratios (HRs) and 95% confidential intervals (CIs). Variables with *P* < 0.1 in univariate analysis and/or clinically important parameters (demographics, comorbidities, and drug use) were included for the final multivariate model. To investigate associations of additional patient characteristics with risk of death and the robustness of HRs to adjustment for additional confounders, we performed sensitivity/subset analysis whereby associations were investigated in a subset of patients who were not missing data for confounders. In addition, we performed receiver operating characteristic (ROC) analysis on baseline liver biochemistry to examine predictive ability for COVID‐19 death, reporting the area under the curve (AUC), sensitivity, and specificity.

#### Longitudinal Analysis of Liver Biochemistry in Patients With COVID‐19

We compared liver biochemistry between the groups with and without COVID‐19 at each time point (to examine differences between groups), and we investigated liver biochemistry changes over time by comparing liver biochemistry at subsequent time points to their baseline within each group. Within the group with COVID‐19, we first examined the changing pattern over time of liver biochemistry in subgroups of patients who died and survived by fitting linear regression lines with 95% CIs and reporting Pearson’s correlation coefficients and linear regression significance, as applied by other longitudinal analyses.^(^ ^25^ ^)^ We then used mixed effects models (lme4, sjPlot packages)^(^ ^26^, ^27^ ^)^ to test if the change in liver biochemistry was significant by considering fixed effects (the follow‐up time, subgroup, and subgroup‐by‐time interaction) and random effect intercept for individuals.

## Results

### Identification, Demographics, and Outcomes of Groups With COVID‐19 Compared to Groups Without COVID‐19

We identified 6,311 eligible patients (585 adults with SARS‐CoV‐2 infection and 5,726 without) according to the inclusion/exclusion criteria (Supporting Fig. S1). Based on our 585 patients positive for COVID‐19, we matched a cohort of 1,165 patients negative for COVID‐19. After matching, there were no significant differences in demographics and preexisting comorbidities between the two groups (Table 1; Supporting Table S4). Median duration of follow‐up was 58 (IQR, 14‐104) days in the group with COVID‐19 and 50 (IQR, 20‐78) days in the matched group without COVID‐19, with no significant difference in the monitoring duration of liver biochemistry (interval between first and last available liver biochemistry test dates) between the groups (Table 1). Within the group with COVID‐19, median follow‐up duration was 10 (IQR, 5‐21) days for those who died and 79 (IQR, 36‐115) days for those who survived. Admitting specialties are summarized in Supporting Table S5.

**TABLE 1:** Baseline characteristics and outcomes of patients with and without COVID‐19

Characteristic	Group With COVID‐19 (n = 585)	Group Without COVID‐19 (n = 1,165)	*P* Value
Overall follow‐up duration (median [IQR]), days	58 [14,104]	50 [20, 78]	*P* = 0.002
Duration of liver biochemistry monitoring (median [IQR]), days	38 [7, 79]	37 [10, 67]	*P* = 0.51
Sex (male), n (%)	312 (53.3)	629 (54.0)	0.83
Age at test (median [IQR])	73 [57, 84]	73 [58, 83]	0.76
Ethnicity category, n (%)			0.92
Asian	31 (5.3)	62 (5.3)	
Black	20 (3.4)	35 (3.0)	
Mixed	10 (1.7)	22 (1.9)	
White	415 (70.9)	804 (69.0)	
Other	9 (1.5)	23 (2.0)	
Not stated	100 (17.1)	219 (18.8)	
BMI (median [IQR]), kg/m^2^	26 [23.2, 31.0]	26 [22.7, 30.5]	0.47
BMI category*, *, n (%)			0.31
<18.5 (underweight)	17 (4.3)	40 (4.9)	
18.5‐24.9 (normal weight)	140 (35.8)	311 (37.9)	
25.0‐29.9 (pre‐obesity/overweight)	115 (29.4)	238 (29.0)	
30.0‐34.9 (obesity class I)	77 (19.7)	122 (14.9)	
35.0‐39.9 (obesity class II)	26 (6.6)	73 (8.9)	
≥40.0 (obesity class III)	16 (4.1)	37 (4.5)	
Preexisting comorbidities, n (%)			
Liver disease (any)	17 (2.9)	33 (2.8)	1
Chronic viral hepatitis	5 (0.9)	5 (0.4)	0.317
Alcoholic liver disease	5 (0.9)	5 (0.4)	0.317
Hepatic failure, not elsewhere classified	3 (0.5)	1 (0.1)	0.112
Fibrosis and cirrhosis of liver	7 (1.2)	10 (0.9)	0.606
Other inflammatory liver diseases	3 (0.5)	5 (0.4)	1
Other diseases of liver	4 (0.7)	24 (2.1)	0.041
Liver disorders in diseases classified elsewhere	0 (0.0)	1 (0.1)	1
DM	88 (15.0)	175 (15.0)	1
HTN	179 (30.6)	349 (30.0)	0.83
CHD	59 (10.1)	113 (9.7)	0.86
CKD	56 (9.6)	107 (9.2)	0.86
Cancer	41 (7.0)	88 (7.6)	0.75
Outcomes			
Death, n (%)	157 (26.8)	139 (11.9)	<0.001
ICU admission, n (%)	71 (12.1)	50 (4.3)	<0.001
Used invasive ventilation in ICU, n (%)	47 (8.0)	29 (2.5)	<0.001
Baseline liver biochemistry^†^			
ALT >ULN, n (%)	102 (20.7)	142 (14.6)	0.004
ALT (median [IQR]), IU/L	25 [16, 40]	19 [13, 32]	<0.001
ALP >ULN, n (%)	98 (19.8)	213 (21.6)	0.47
ALP (median [IQR]), IU/L	85 [66, 114]	90 [71, 124]	0.005
BR >ULN, n (%)	29 (5.9)	127 (13.0)	<0.001
BR (median [IQR]), µmol/L	9 [6, 13]	10 [7, 16]	<0.001
Albumin <LLN, n (%)	291 (58.7)	346 (35.0)	<0.001
Albumin (median [IQR]), g/L	30 [27, 34]	34 [29, 37]	<0.001
Baseline blood clotting tests^‡^, (median [IQR])			
Prothrombin time, seconds	10.9 [10.4, 11.4]	10.9 [10.4, 11.8]	0.093
APTT, seconds	24.8 [23.0, 27.7]	24.4 [22.6, 27.1]	0.014
INR	1.0 [1.0, 1.1]	1.0 [1.0, 1.1]	0.073
Baseline renal function tests			
Creatinine (median [IQR]), µmol/L	82 [65, 111]	81 [65, 117]	0.71
Elevated creatinine, n (%)	151 (29.9)	321 (31.3)	0.63
Urea (median [IQR]), mmol/L	6.3 [4.3, 9.8]	6.2 [4.5, 9.9]	0.64
eGFR (median [IQR]), mL/minute/1.73 m^2^	76 [49, >90]	76 [47, >90]	0.65
eGFR <90 mL/minute/1.73 m^2^, n (%)	369 (73.2)	746 (72.6)	0.86
Other tests at baseline, (median [IQR])			
CRP, mg/L	78 [27, 148]	20 [4, 93]	<0.001
Platelets, × 10^9^/L	216 [160, 281]	245 [195, 311]	<0.001
Lymphocytes, × 10^9^/L	0.9 [0.6, 1.3]	1.2 [0.8, 1.8]	<0.001
Baseline vital signs^§^, (median [IQR])			
Temperature tympanic, °C	37 [36.5, 38]	36.5 [36, 37]	<0.001
Heart rate, bpm	86 [76, 97]	81 [72, 95]	<0.001
Oxygen saturation, %	95 [94, 97]	96 [95, 98]	<0.001
Respiratory rate	20 [18, 22]	18 [17, 19]	<0.001
Diastolic blood pressure, mm Hg	70 [64, 78]	72 [64, 81]	0.076
Systolic blood pressure, mm Hg	129 [118, 143]	133 [121, 149]	<0.001

Data are the median [IQR] or number (%) unless otherwise indicated. For categorical variables, Fisher's exact test was performed for comparison on cells with small counts (<5), otherwise chi‐square test was used. For continuous variables, Wilcoxon test was used for comparison due to non‐normality. *P* < 0.05 is considered significant.

* 194 versus 344 missing on BMI data; BMI categories were reported based on WHO classification.

^†^ 93 versus 191 patients with versus without COVID‐19 did not have baseline data available on all four liver biochemistries; in detail, 93 versus 191 missing for ALT; 91 versus 180 missing for ALP; 93 versus 191 missed data for BR; 89 versus 177 missing for albumin.

^‡^ 158 versus 303 patients with versus without COVID‐19 did not have baseline data available on blood clotting tests.

^§^ 162 versus 324 patients with versus without COVID‐19 did not have baseline data available on vital signs.

Abbreviations: APTT, activated partial thromboplastin time; bpm, beats per minute; eGFR, estimated glomerular filtration rate.

**FIG. 1:**
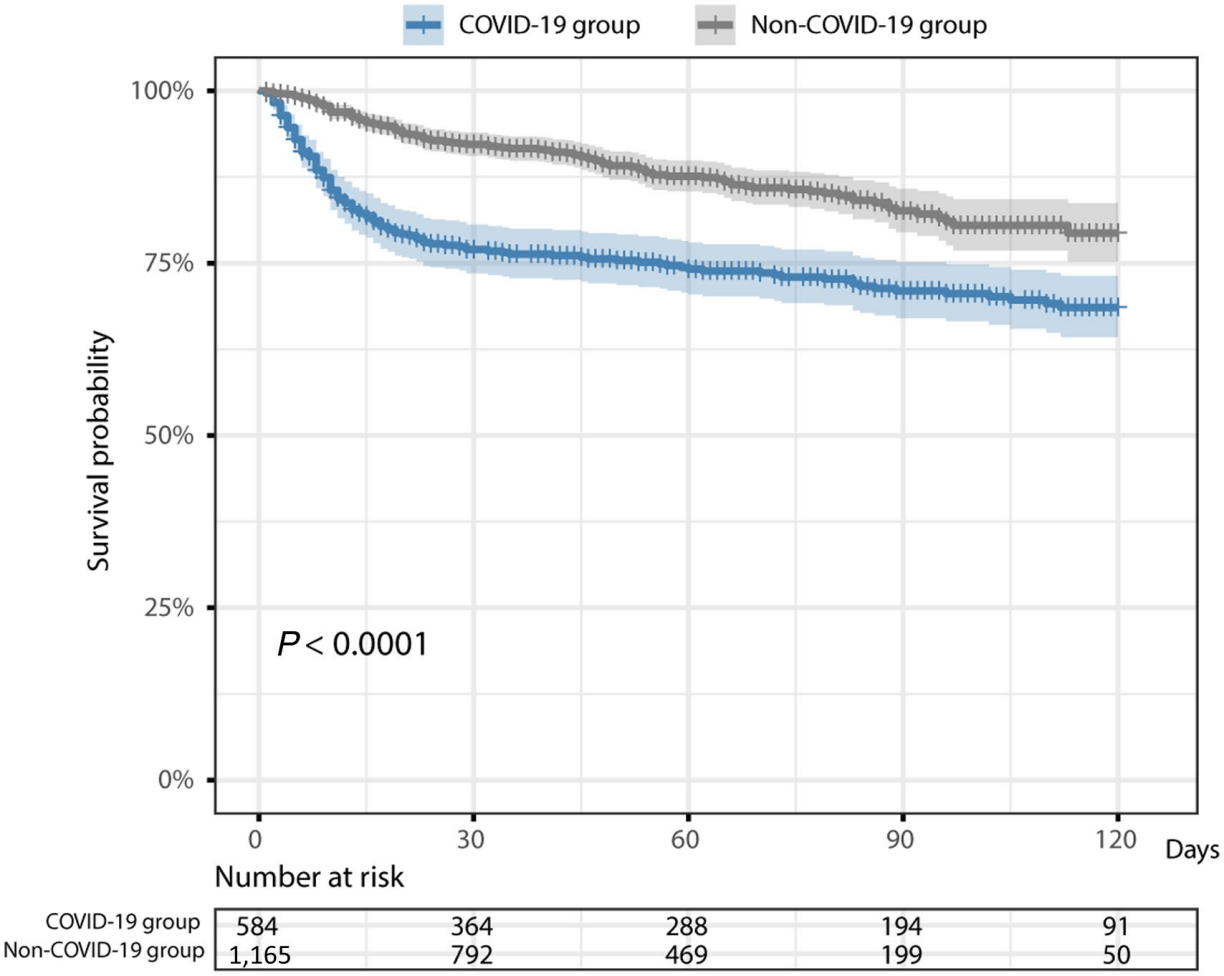
K‐M curves for the comparison of time to death in the group with COVID‐19 vs. the group without COVID‐19 following the SARS‐CoV‐2 RT‐PCR test. The death date for 1 patient in the group with COVID‐19 was missing. The *P* value is based on the log‐rank test.

In patients with COVID‐19, 26.8% (157/585) died compared to 11.9% (139/1,165) in the group without COVID‐19 (*P* < 0.001). The group with COVID‐19 had a higher rate of ICU admission (12.1% vs. 4.3%, *P* < 0.001) and a higher rate of invasive ventilation use (8% vs. 2.5%, *P* < 0.001) compared to the group without COVID‐19 (Table 1). The K‐M estimated probability of surviving >30 days after the SARS‐CoV‐2 RT‐PCR test was 77% for patients with COVID‐19 versus 92% for patients without COVID‐19 (Fig. 1).

### Assessment of Liver Biochemistry in the Group With COVID‐19 Compared to the Group Without COVID‐19

Baseline liver biochemistry was available in 492 patients with COVID‐19 and 974 patients without COVID‐19. The median time interval between consecutive liver biochemistry measurements was 1 (IQR, 1‐4) day and 2 (IQR, 1‐7) days (*P* < 0.001) for the groups with and without COVID‐19, respectively.

Overall, the group with COVID‐19 had a significantly higher proportion of patients with one or more deranged liver biochemistry tests at baseline compared to the group without COVID‐19 (72.6% vs. 55.5%, *P* < 0.001) (Supporting Table S6). At baseline, the group with COVID‐19 had a significantly higher median ALT value (25 IU/L vs. 19 IU/L, *P* < 0.001) and a higher proportion of patients with ALT >ULN (20.7% vs. 14.6%, *P* = 0.004) than the group without COVID‐19 (Table 1). The group with COVID‐19 also had a lower median albumin (30 g/L vs. 34 g/L, *P* < 0.001), lower platelets (216 × 10^9^/L vs. 245 × 10^9^/L, *P* < 0.001), lower lymphocytes (0.9 × 10^9^/L vs. 1.2 × 10^9^/L, *P* < 0.001), and significantly higher C‐reactive protein (CRP; 78 mg/L vs. 20 mg/L, *P* < 0.001) compared to the group without COVID‐19. Baseline vital signs were also more deranged in the group with COVID‐19, while renal function was preserved (Table 1).

**FIG. 2:**
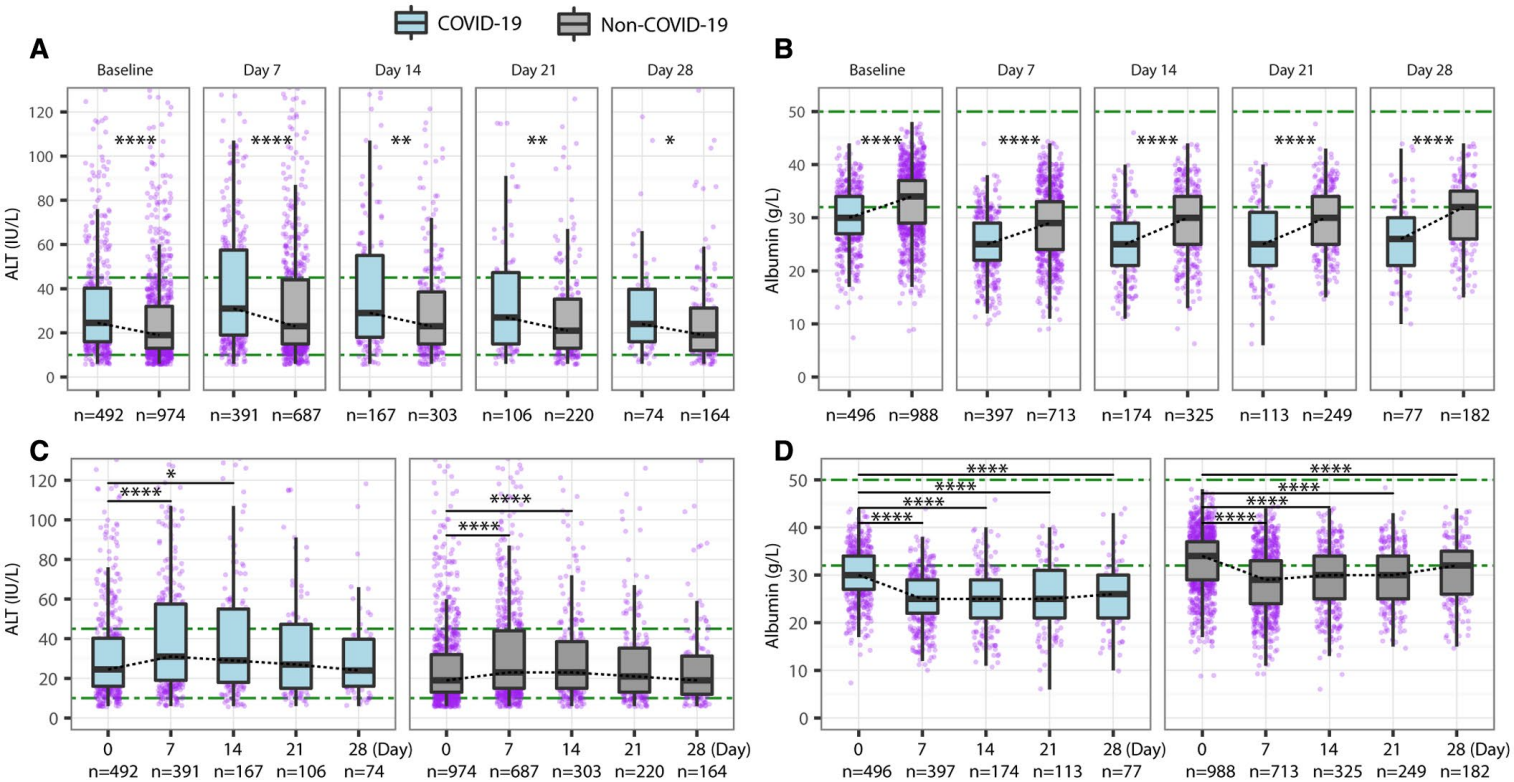
Comparison of ALT and albumin between hospitalized adults with and without COVID‐19 matched for age, sex, ethnicity, and preexisting comorbidities. (A) ALT comparison at baseline and at 7, 14, 21, and 28 days. (B) Albumin comparison at baseline and at 7, 14, 21, and 28 days. (C) ALT changes over time in the groups with and without COVID‐19. (D) Albumin changes over time in the groups with and without COVID‐19. Green dash‐dotted lines indicate the LLNs and ULNs. The box represents the median and interquartile range, and the whiskers represent the adjacent values, which are within 1.5 times the interquartile range. The purple dots represent the observation values. *P < 0.05, **P < 0.01, ****P < 0.0001.

Over follow‐up, the group with COVID‐19 had more deranged liver biochemistries, with a higher median peak ALT (34 IU/L vs. 26 IU/L, *P* < 0.001), a higher proportion with peak ALT >ULN (37.9% vs. 27.7%, *P* < 0.001), a lower median nadir albumin (26 g/L vs. 29 g/L, *P* < 0.001), and a higher prevalence of hypoalbuminemia (79.0% vs. 59.5%, *P* < 0.001) compared to the group without COVID‐19 (Supporting Table S6). Patients with COVID‐19 also had significantly higher median ALT and lower median albumin values at time points throughout follow‐up (7, 14, 21, and 28 days) compared to the group without COVID‐19 (all *P* < 0.05) (Fig. 2A,B). In the group with COVID‐19, median ALT increased at 7 and 14 days compared to baseline (both *P* < 0.05) (Fig. 2C) and median albumin decreased at 7 days (*P* < 0.0001) and remained at low levels at subsequent time points (Fig. 2D). We did not identify differences in ALP and BR over time between these groups other than at baseline or at 7 days follow‐up (Supporting Fig. S2).

Baseline vital signs were recorded for 423 patients with COVID‐19 and used to stratify disease severity, with 65 severe/critical COVID‐19 cases and 358 mild/moderate cases. As expected, a higher rate of death was observed in severe/critical cases compared to mild/moderate cases (43.1% vs. 27.1%, *P* = 0.01). A higher proportion (31% vs. 19%, *P* = 0.04) in the severe/critical group had elevated ALT at baseline. However, there were no other significant differences in liver biochemistry between mild/moderate and serve/critical cases at baseline or during follow‐up (Table 2).

**TABLE 2:** Comparison of demographics, comorbidities, outcomes, and baseline and peak/nadir liver biochemistries between COVID‐19 subgroups stratified by disease severity

Characteristic	Mild/Moderate COVID‐19 Cases (n = 358)*, *	Severe/Critical COVID‐19 Cases (n = 65)^†^	*P* Value
Sex (male), n (%)	198 (55.3)	31 (47.7)	0.32
Age at test (median [IQR])	74 [59, 84]	71 [56, 84]	0.54
Ethnicity category, n (%)			0.44
Asian	19 (5.3)	1 (1.5)	
Black	11 (3.1)	2 (3.1)	
Mixed	5 (1.4)	3 (4.6)	
White	260 (72.6)	48 (73.8)	
Other	4 (1.1)	1 (1.5)	
Not stated	59 (16.5)	10 (15.4)	
BMI (median [IQR]), kg/m^2^	26.9 [23.6, 30.9]	25.7 [21.6, 32.0]	0.58
BMI category^‡^, n (%)			0.47
<18.5 (underweight)	9 (3.4)	2 (5.4)	
18.5‐24.9 (normal weight)	90 (34.0)	15 (40.5)	
25.0‐29.9 (pre‐obesity/overweight)	85 (32.1)	6 (16.2)	
30.0‐34.9 (obesity class I)	52 (19.6)	10 (27.0)	
35.0‐39.9 (obesity class II)	18 (6.8)	3 (8.1)	
≥40.0 (obesity class III)	11 (4.2)	1 (2.7)	
Preexisting comorbidities, n (%)			
Liver disease	10 (2.8)	2 (3.1)	1
DM	52 (14.5)	7 (10.8)	0.54
HTN	109 (30.4)	13 (20.0)	0.12
CHD	36 (10.1)	5 (7.7)	0.72
CKD	33 (9.2)	3 (4.6)	0.33
Cancer	27 (7.5)	3 (4.6)	0.56
Outcomes			
Death, n (%)	97 (27.1)	28 (43.1)	0.012
ICU admission, n (%)	37 (10.3)	18 (27.7)	<0.001
Used invasive ventilation in ICU, n (%)	23 (6.4)	14 (21.5)	<0.001
Baseline liver biochemistry			
ALT >ULN, n (%)	66 (19.0)	20 (31.2)	0.041
ALT (median [IQR]), IU/L	24.0 [17.0, 39.0]	29.5 [18.8, 50.2]	0.063
ALP >ULN, n (%)	71 (20.4)	10 (15.6)	0.48
ALP (median [IQR]), IU/L	84.0 [66.0, 116.2]	80.0 [60.8, 104.0]	0.19
BR >ULN, n (%)	21 (6.1)	2 (3.1)	0.52
BR (median [IQR]), µmol/L	9.0 [6.0, 14.0]	10.0 [7.0, 13.2]	0.44
Albumin <LLN, n (%)	201 (57.8)	39 (60.9)	0.74
Albumin (median [IQR]), g/L	30.5 [27.0, 34.0]	30.5 [26.8, 33.2]	0.52
Peak/nadir liver biochemistry			
ALT (median [IQR]), IU/L	37.0 [23.0, 65.8]	39.0 [26.0, 97.0]	0.39
ALT >ULN, n (%)	148 (41.3)	29 (44.6)	0.72
ALP (median [IQR]), IU/L	108.0 [81.0, 158.0]	95.0 [75.0, 152.0]	0.16
ALP >ULN, n (%)	133 (37.2)	18 (27.7)	0.19
BR (median [IQR]), µmol/L	12.0 [8.0, 17.0]	13.0 [9.0, 16.0]	0.67
BR >ULN, n (%)	52 (14.5)	5 (7.7)	0.20
Albumin (median [IQR]), g/L	25.0 [21.0, 29.0]	25.0 [21.0, 28.0]	0.55
Albumin <LLN, n (%)	307 (85.8)	57 (87.7)	0.83

Data are the median [IQR] or number (%) unless otherwise indicated. For categorical variables, Fisher's exact test was performed for comparison on cells with small counts (<5), otherwise chi‐square test was used. For continuous variables, Wilcoxon test was used for comparison due to non‐normality. *P* < 0.05 is considered significant.

* Mild/Moderate: baseline respiratory rate ≤30 breaths/minute and SpO_2_ ≥90%.

^†^ Severe/Critical: respiratory rate >30 breaths/minute, or SpO_2_ <90%.

^‡^ 93 in mild/moderate cases versus 28 in severe/critical cases missing on BMI data; BMI categories were reported based on WHO classification.

Abbreviation: SpO_2_, oxygen saturation.

Compared to the group without COVID‐19, both the subgroups with mild/moderate COVID‐19 and severe/critical COVID‐19 had a significantly higher prevalence of abnormal liver biochemistry both at baseline and during follow‐up, with significantly higher rates of mortality, ICU admission rate, and the proportion requiring invasive ventilation (Supporting Tables S7 and S8). These results were consistent with those from the comparison between the whole group with COVID‐19 and the group without COVID‐19.

### Demographics, Comorbidities, and Liver Biochemical Characteristics Associated With Mortality in Patients With COVID‐19

In the group with COVID‐19, patients who died were significantly older than those who survived (median, 82 years vs. 66 years, respectively, *P* < 0.001); significantly more likely to have preexisting comorbidities, including liver disease, DM, CHD, and cancer; significantly more likely to have baseline hypoalbuminemia (72.6% vs. 52.9%, *P* < 0.001); and more likely to have nadir albumin below the lower limit of the normal (LLN) range during follow‐up (96.2% vs. 72.7%, *P* < 0.001) (Table 3). In patients who died, baseline ALP was higher (*P* = 0.007) and peak BR was more likely to be abnormal compared to those who survived (*P* = 0.016; Supporting Table S9). Equivalent data for the group without COVID‐19 are provided in Supporting Table S10.

**TABLE 3:** Demographics, preexisting comorbidities, and baseline and peak/nadir liver biochemistry of patients with COVID‐19 who survived and died

Characteristic	Survived (n = 428)	Died (n = 157)	*P* Value
Sex, male, n (%)	221 (51.6)	91 (58.0)	0.21
Age at test (median [IQR]), years	66 [54, 80]	82 [75, 89]	<0.001
Age ≥75 years, n (%)	158 (36.9)	120 (76.4)	<0.001
Ethnicity category, n (%)			
Asian	29 (6.8)	2 (1.3)	0.015
Black	18 (4.2)	2 (1.3)	0.14
Mixed and other	17 (4.0)	2 (1.3)	0.17
White	287 (67.1)	128 (81.5)	0.001
Not stated	77 (18.0)	23 (14.6)	0.41
BMI (median [IQR]), kg/m^2^	27 [23.4, 31.3]	25 [22, 30.2]	0.076
BMI category*, n (%)			
<18.5 (underweight)	10 (3.5)	7 (6.7)	0.28
18.5‐24.9 (normal weight)	98 (34.3)	42 (40.0)	0.35
25.0‐29.9 (pre‐obesity/overweight)	87 (30.4)	28 (26.7)	0.55
30.0‐34.9 (obesity class I)	57 (19.9)	20 (19.0)	0.96
35.0‐39.9 (obesity class II)	21 (7.3)	5 (4.8)	0.49
≥40.0 (obesity class III)	13 (4.5)	3 (2.9)	0.65
Used invasive ventilation in ICU, n (%)	41 (9.6)	6 (3.8)	0.036
ICU admission, n (%)	59 (13.8)	12 (7.6)	0.061
Preexisting comorbidities, n (%)			
Liver disease	6 (1.4)	11 (7.0)	0.001
DM	53 (12.4)	35 (22.3)	0.005
HTN	121 (28.3)	58 (36.9)	0.055
CHD	35 (8.2)	24 (15.3)	0.018
CKD	36 (8.4)	20 (12.7)	0.16
Cancer	24 (5.6)	17 (10.8)	0.045
≥1 liver biochemistry abnormal at baseline^†^, n (%)	235 (67.9)	122 (83.6)	0.001
≥1 liver biochemistry abnormal at baseline (excluding albumin), n (%)	123 (35.5)	55 (37.7)	0.73
Baseline ALT (median [IQR]), IU/L	25 [16, 43]	23 [16, 36]	0.11
Baseline ALT categories^‡^, n (%)			
normal	267 (77.2)	123 (84.2)	0.099
>1‐2 ULN	49 (14.2)	15 (10.3)	0.31
>2‐3 ULN	16 (4.6)	5 (3.4)	0.72
>3 ULN	14 (4.0)	3 (2.1)	0.40
Baseline albumin (median [IQR]), g/L	31 [28, 34]	28 [25, 32]	<0.001
Baseline albumin (<LLN), n (%)	185 (52.9)	106 (72.6)	<0.001
≥1 peak/nadir liver biochemistry abnormal, n (%)	352 (82.2)	153 (97.5)	<0.001
≥1 peak liver biochemistry abnormal (excluding albumin), n (%)	238 (55.6)	91 (58.0)	0.68
Peak ALT (median [IQR]), IU/L	37 [20, 71]	32 [19, 52]	0.07
Peak ALT categories, n (%)			
normal	252 (58.9)	111 (70.7)	0.012
>1‐2 ULN	93 (21.7)	22 (14.0)	0.0496
>2‐3 ULN	28 (6.5)	11 (7.0)	0.99
>3 ULN	55 (12.9)	13 (8.3)	0.17
Nadir albumin (median [IQR]), g/L	27 [22, 32]	23 [19, 27]	<0.001
Nadir albumin (<LLN)^§^, n (%)	311 (72.7)	151 (96.2)	<0.001

For categorical variables, Fisher's exact test was performed for comparison on cells with small counts (<5), otherwise chi‐square test was used. For continuous variables, Wilcoxon test was used for comparison due to non‐normality. *P* < 0.05 is considered significant.

* BMI categories were reported based on WHO classification.

^†^ 82 versus 11 in survived subgroup versus died subgroup did not have all liver biochemistry baseline data available.

^‡^ 82 versus 11 in survived subgroup versus died subgroup missing baseline ALT.

^§^ 78 versus 11 in survived subgroup versus died subgroup missing baseline albumin.

**FIG. 3:**
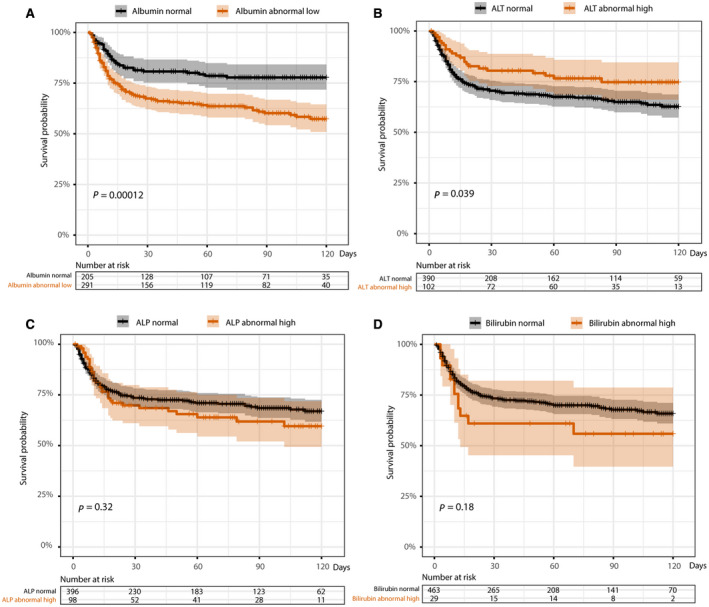
Survival K‐M curves stratified by baseline liver biochemistry at the time of testing positive for SARS‐CoV‐2. (A) Normal and low baseline albumin. (B) Normal and elevated baseline ALT. (C) Normal and elevated baseline ALP. (D) Normal and elevated baseline BR. *P* values are based on the log‐rank test. *P* < 0.05 is considered significant. The death date of 1 patient was missing.

Survival curves and log‐rank tests (unadjusted for relevant confounders) within the group with COVID‐19 showed that hypoalbuminemia compared to normal albumin at baseline was significantly associated with an increase in mortality (Fig. 3A). Surprisingly an elevated baseline ALT compared to a normal ALT at baseline was significantly associated with an increase in survival (Fig. 3B). An elevated ALP or BR at baseline was not significantly associated with lower survival probability over time (Fig. 3C,D). In addition, for the subset of patients with COVID‐19 who had prothrombin time measured, elevation of this parameter (and of international normalized ratio [INR]) at baseline was significantly associated with a lower survival probability (Supporting Fig. S3).

In multivariate analysis (fully adjusted for demographics, comorbidities, and prescribed drug use before baseline) for the group with COVID‐19, those with hypoalbuminemia (i.e., <32 g/L) at baseline had a 1.83‐fold increased hazards of death compared to those with normal baseline albumin (adjusted HR, 1.83; 95% CI, 1.25‐2.67). Those aged ≥75 years had a ~4‐fold increased hazards of death compared to those aged <75 years (adjusted HR, 3.96; 95% CI, 2.59‐6.04), and those with preexisting liver disease had a ~3‐fold increased hazards of death than those without preexisting liver disease (adjusted HR, 3.37; 95% CI, 1.58‐7.16) (Table 4). However, we found no significant association between baseline ALT and hazards of death (adjusted HR, 0.86; 95% CI, 0.53‐1.38; *P* = 0.53) in the fully adjusted analysis (Table 4). A 1‐unit (1 g/L) decrease in albumin at baseline was significantly associated with a 5% increase in hazards of death (adjusted HR, 1.05; 95% CI, 1.02‐1.09), while an age increase by 10 years was associated with an 82% increase in hazards of death (adjusted HR, 1.82; 95% CI, 1.56‐2.13) (Supporting Table S11). Similarly, a 1‐unit decrease in nadir albumin during follow‐up was significantly associated with a 7% increase in hazards of death (adjusted HR, 1.07; 95% CI, 1.04‐1.10) (Supporting Table S12). In the group with COVID‐19, baseline albumin was significantly negatively correlated with age in those that survived (R = −0.264, *P* < 0.0001) but not in those who died (R = −0.027, *P* = 0.75) (Supporting Fig. S4).

**TABLE 4:** Univariate and multivariate Cox proportional hazards models investigating associations of baseline liver biochemistry derangement with death among adults with confirmed COVID‐19

Variables	Univariate Analysis		Multivariate Analysis	
	Crude HR (95% CIs)	*P* value	Adjusted HR (95% CIs)	*P* Value
Age ≥75 years	4.39 (3.03‐6.36)	<0.001	3.96 (2.59‐6.04)	<0.001
Sex (male)	1.24 (0.91‐1.71)	0.18	1.21 (0.86‐1.71)	0.28
Ethnicity (white)	1.87 (1.25‐2.8)	0.002	1.13 (0.71‐1.8)	0.61
Baseline ALT (>ULN)	0.63 (0.4‐0.98)	0.04	0.86 (0.53‐1.38)	0.53
Baseline ALP (>ULN)	1.21 (0.83‐1.78)	0.33		
Baseline BR (>ULN)	1.5 (0.83‐2.7)	0.18		
Baseline albumin (<LLN)	2.01 (1.4‐2.9)	<0.001	1.83 (1.25‐2.67)	0.002
Preexisting liver disease	2.66 (1.44‐4.9)	0.002	3.37 (1.58‐7.16)	0.002
Preexisting DM	1.7 (1.16‐2.48)	0.006	1.22 (0.77‐1.93)	0.39
Preexisting HTN	1.33 (0.96‐1.84)	0.085	0.7 (0.44‐1.11)	0.13
Preexisting CHD	1.72 (1.11‐2.65)	0.015	1.16 (0.67‐2.02)	0.59
Preexisting CKD	1.34 (0.83‐2.17)	0.23	0.84 (0.46‐1.51)	0.56
Preexisting Cancer	1.71 (1.04‐2.84)	0.036	1.06 (0.58‐1.93)	0.85

Analysis were performed on the 492 patients with COVID‐19 who had baseline liver biochemistry data (survived vs. died, 346 vs. 146); the cut‐off age was based on the median age in the cohort. Liver biochemistry variables with P < 0.1, demographics, and comorbidities in univariate analysis were included for multivariate analysis. In multivariate analysis, HRs were fully adjusted for drug use before baseline (including antiviral drugs, antibiotics, anticoagulants, acetaminophen, immunosuppressants, statins) to reduce confounding effects.

**FIG. 4:**
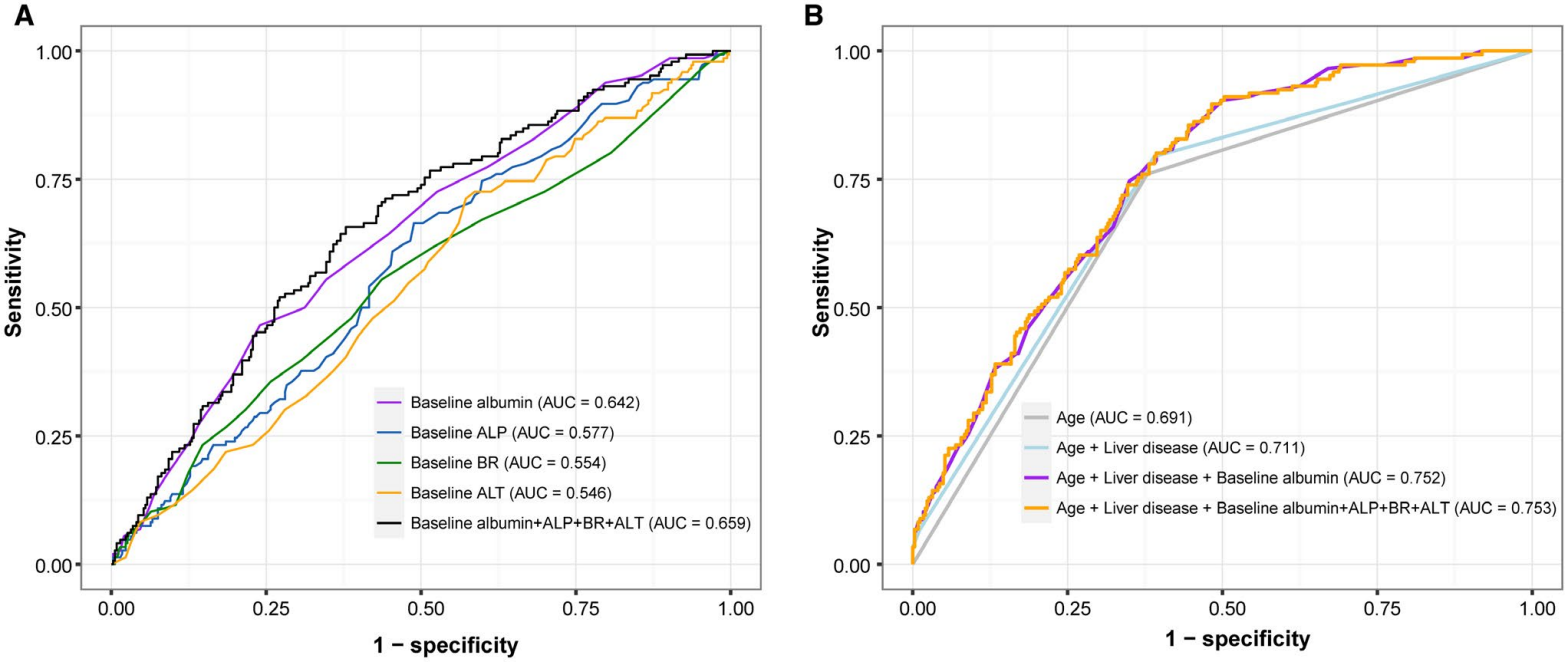
ROC curves of predicting death. (A) Baseline liver biochemistry. (B) Baseline liver biochemistry and other identified predictors of mortality. Age (≥75 years or <75 years) and liver disease (yes/no) are used as binary variables, which were identified from the multivariate Cox proportional hazards model in Table 4. Baseline albumin + ALP + BR + ALT in the figure indicates including all baseline liver biochemistry parameters in the ROC analysis.

ROC analysis further demonstrated the prognostic value of baseline albumin for COVID‐19 death, which had higher overall performance (AUC = 0.642) compared to that of baseline ALP, BR, or ALT (Fig. 4A). The combination of baseline liver biochemistry parameters did not significantly improve the performance (AUC = 0.659, *P* = 0.08) (Fig. 4A; Supporting Table S13). Considering the other predictors (age and liver disease) identified from the multivariate model, we found adding albumin can further improve prediction of death (AUC significantly improved from 0.711 to 0.752, *P* = 0.002) (Fig. 4B; Supporting Table S14). Adding other baseline liver biochemistry parameters did not make further significant improvement (Fig. 4B).

For the subset of patients with COVID‐19 who had BMI data (survived vs. died, 246 vs. 96), HRs did not change materially after additional adjustment for BMI in the multivariate analysis (Supporting Table S15). Baseline albumin was weakly correlated with BMI both in those who died and survived (R = 0.08, *P* = 0.42 vs. R = 0.099, *P* = 0.12, respectively) (Supporting Fig. S5).

### Longitudinal Assessment of Liver Biochemistry Patterns in Patients Who Died With COVID‐19 Compared to Those Who Survived.

**FIG. 5:**
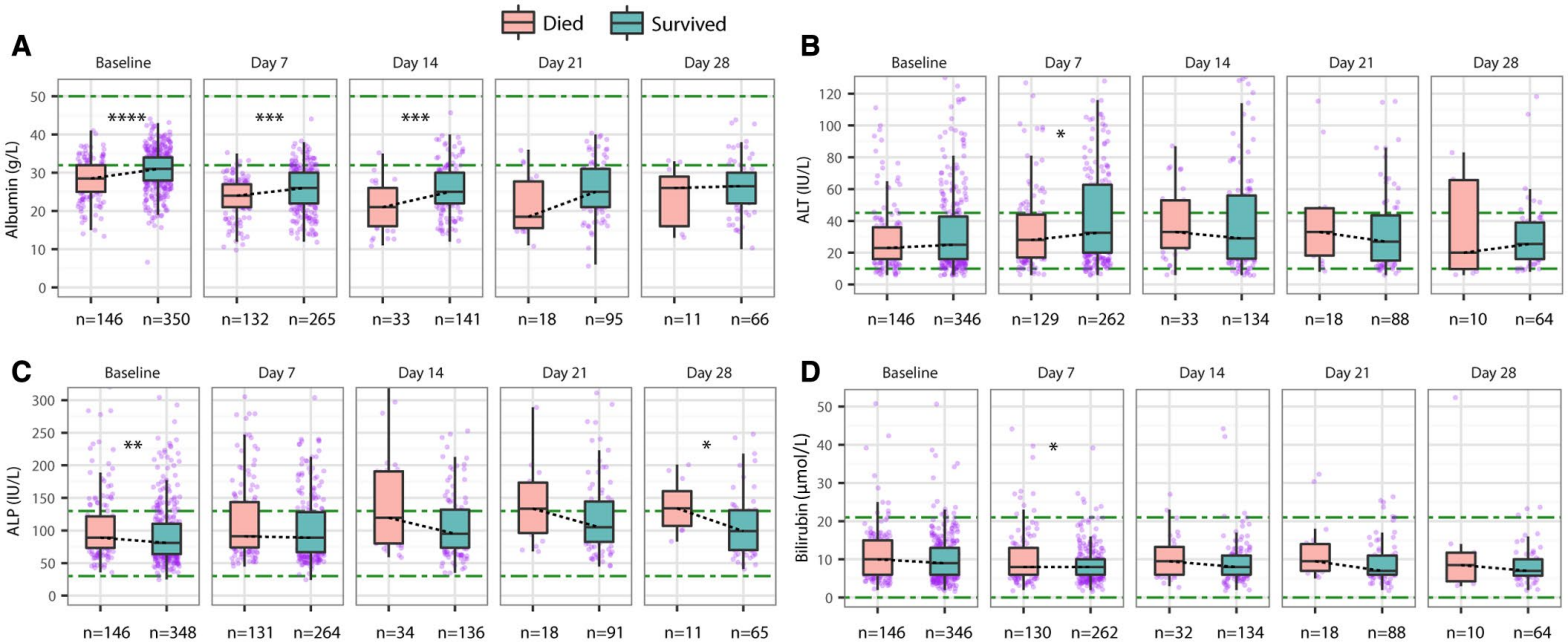
Comparison of liver biochemistry between patients with COVID‐19 who died during follow‐up and who survived to the end of follow‐up. (A) Albumin comparison at baseline and 7, 14, 21, and 28 days; (B) ALT comparison at baseline and 7, 14, 21, and 28 days. (C) ALP comparison at baseline and 7, 14, 21, and 28 days. (D) BR comparison at baseline and 7, 14, 21, and 28 days. Green dash‐dotted lines indicate LLNs and the ULNs. The box represents the median and interquartile range, and the whiskers represent the adjacent values, which are within 1.5 times the interquartile range. The purple dots represent the observation values. *P < 0.05, **P < 0.01, ***P < 0.001, ****P < 0.0001.

Within the group with COVID‐19, patients who died during follow‐up had significantly lower median albumin at baseline and at 7 and 14 days after a positive SARS‐CoV‐2 RT‐PCR compared to the patients who survived (all *P* < 0.001) (Fig. 5A). There was no significant difference in ALT at any time point other than 7 days between those who died and survived (Fig. 5B). For patients who died, ALP was higher at baseline and 28 days and BR was higher at 7 days compared to those who survived (all *P* < 0.05) (Fig. 5C,D).

**FIG. 6:**
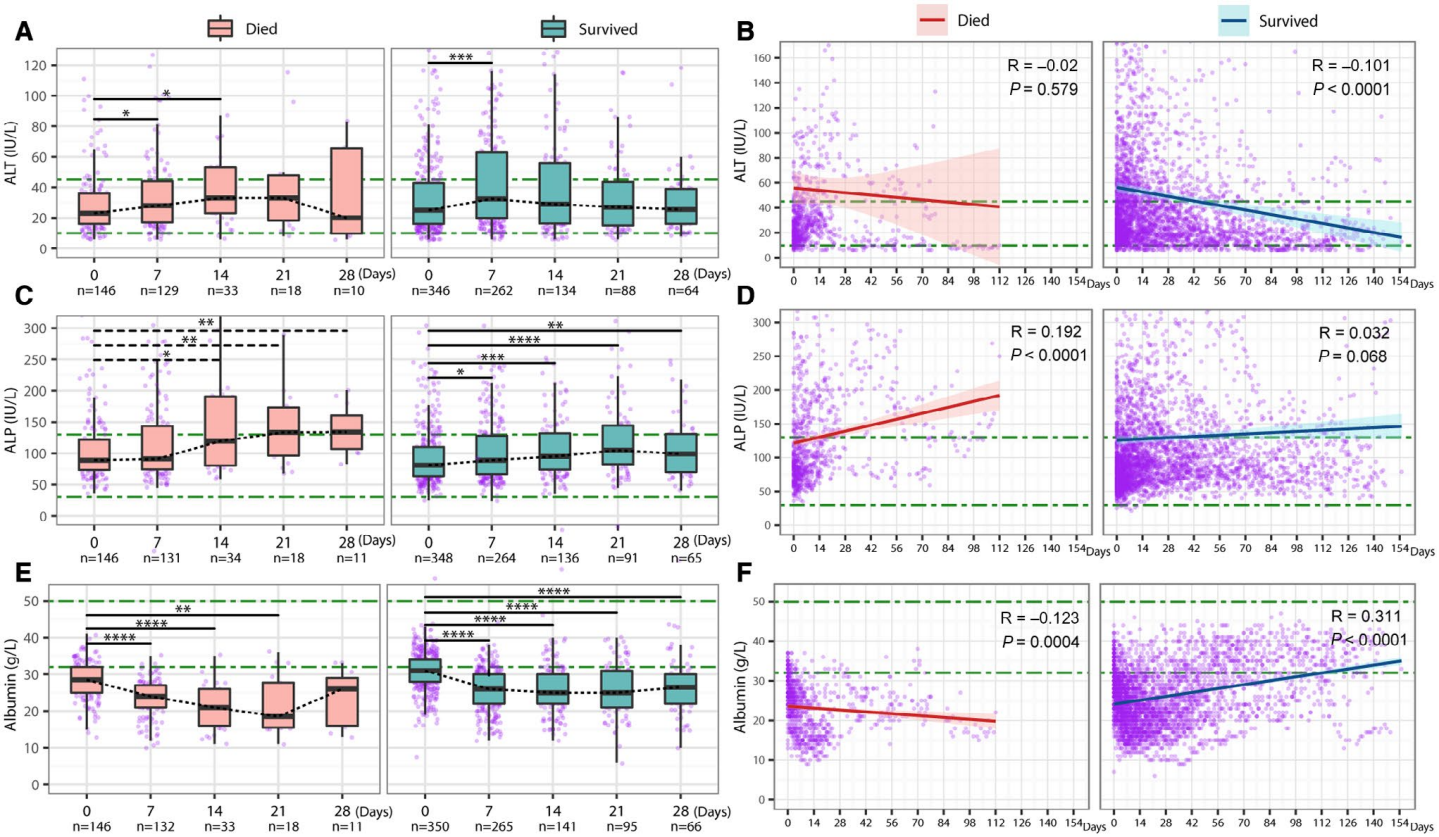
Longitudinal changes of ALT, ALP, and albumin over time of patients with COVID‐19 stratified by death during follow‐up. (A) ALT at baseline and 7, 14, 21, and 28 days. (B) Changing trend over time (112 vs. 155 days) of ALT by linear regression line fitting with 95% CI. (C) ALP at baseline, 7, 14, 21, and 28 days; (D) Changing trend over time (112 vs. 155 days) of ALP by linear regression line fitting with 95% CI. (E) Albumin at baseline and 7, 14, 21, and 28 days. (F) Changing trend over time (112 vs. 155 days) of albumin by linear regression line fitting with 95% CI. Green dash‐dotted lines indicate LLNs and ULNs. The box represents the median and interquartile range, and the whiskers represent the adjacent values, which are within 1.5 times the interquartile range. The purple dots represent the observation values. *P < 0.05, **P < 0.01, ***P < 0.001, ****P < 0.0001. R is the Pearson’s correlation coefficient and *P* is the linear regression significance.

In the group with COVID‐19, ALT increased during the first 2 weeks (*P* < 0.05) and remained elevated in patients who died (R = −0.020, *P* = 0.6), while in patients who survived, ALT decreased from 7 days onward, with a significantly decreasing trend to normalization (R = −0.101, *P* < 0.0001) (Fig. 6A,B). ALP significantly increased over time in those who died (R = 0.192, *P* < 0.0001), compared to those who survived (R = 0.032, *P* = 0.068) (Fig. 6C,D). Albumin decreased significantly in both groups during the first 7 days and continued to decline in patients who died (R = −0.123, *P* = 0.0004), but patients who survived demonstrated an upward trend in albumin, especially for follow‐up >1 month (R = 0.311, *P* < 0.0001) (Fig. 6E,F). BR decreased throughout follow‐up in both subgroups, but the decline was not significant (R = −0.052, *P* = 0.146; R = −0.013, *P* = 0.483) (Supporting Fig. S6A,B).

Consistently, the mixed effects model also showed that within the subgroup who died, albumin significantly decreased over time (β = −0.12; 95% CI, −0.14 to −0.10; *P* < 0.001) while ALP significantly increased over time (β = 0.50; 95% CI, 0.29‐0.70; *P* < 0.001), but the change in ALT or BR was not significant (*P* = 0.817, *P* = 0.489, respectively) (Supporting Table S16). Considering the interaction between subgroup and time, the mixed effects model further revealed that (a) changes in albumin were significantly different over time between subgroups that died vs. survived (*P* < 0.001), with a decrease in the subgroup that died (β_time + 1 × β_interaction = −0.11) and an increase in the subgroup that survived (β_time + 0 × β_interaction = 0.08) based on the coefficients of time and interaction; (b) changes in ALP were also significantly different over time for the subgroups (*P* = 0.005), increasing in the subgroup that died (β_time + 1 × β_interaction = 0.50) while decreasing in the subgroup that survived (β_time + 0 × β_interaction = −0.07); (c) changes in ALT (*P* = 0.126) or BR (*P* = 0.356) were insignificantly different over time for the subgroups that died and survived (Table 5).

**TABLE 5:** Changes in liver biochemistries over the time assessed by the linear mixed effects model for patients with COVID‐19 between groups (Died vs. Survived)

Parameter	Coefficient (β)	95% CI	*P* Value
Albumin			
(Intercept)	27.72	(27.16, 28.29)	<0.001
Group	−1.68	(−2.76, −0.60)	0.002
Time	0.08	(0.07, 0.08)	<0.001
Interaction (group × time)	−0.19	(−0.2, −0.17)	<0.001
ALP			
(Intercept)	110.49	(101.83, 119.16)	<0.001
Group	5.34	(−11.37, 22.04)	0.531
Time	−0.07	(−0.17, 0.02)	0.133
Interaction (group × time)	0.57	(0.17, 0.97)	0.005
ALT			
(Intercept)	47.9	(41.04, 54.76)	<0.001
Group	−1.66	(−14.95, 11.63)	0.807
Time	−0.19	(−0.30, −0.08)	0.001
Interaction (group × time)	0.34	(−0.09, 0.77)	0.126
BR			
(Intercept)	10.56	(9.60, 11.52)	<0.001
Group	2.17	(0.32, 4.01)	0.021
Time	−0.02	(−0.03, −0.01)	<0.001
Interaction (group × time)	0.02	(−0.02, 0.07)	0.356

Group = {0, 1}, where 0 indicates survived, 1 indicates died. *P* < 0.05 is considered significant.

Among patients with COVID‐19 who had ≥2 longitudinal data points for ALT, ALP, BR, and albumin, 468 had at least one liver biochemistry derangement during follow‐up. Among them, 26.9% (126/468) had normalized by the end of follow‐up, while the remaining 73.1% still had one or more abnormal liver biochemistries at the end of follow‐up (Supporting Table S17).

## Discussion

We used an automatic approach through an established NIHR HIC clinical data collating bioinformatic pipeline^(^ ^22^, ^23^ ^)^ to capture a complete record of relevant clinical and laboratory parameters from hospitalized patients within the time period assessed. This resource allows us to fully analyze liver biochemistry abnormalities and outcomes on a large cohort of patients with COVID‐19 and propensity score‐matched controls without COVID‐19 for the first time. To our knowledge, this is the first study to comprehensively i) conduct longitudinal analyses of liver biochemistry patterns over time in patients with COVID‐19 compared to a matched cohort of patients without COVID‐19 attending hospital in the United Kingdom; ii) investigate whether albumin, in addition to other liver biochemistries, at baseline and during follow‐up is associated with death in patients with COVID‐19; and iii) analyze longitudinal liver biochemistry patterns in patients with COVID‐19 who subsequently die or survive.

The group with COVID‐19 exhibited a ~2‐fold higher death rate with a significantly lower survival probability compared to the group without COVID‐19. Among the group with COVID‐19, a higher proportion of patients had at least one abnormal liver biochemistry compared to the matched group without COVID‐19. Patients with COVID‐19 who died showed a decline in albumin and a greater increase in ALP over time compared to those who survived, and baseline hypoalbuminemia was a significant predictor of death in patients with COVID‐19 on multivariate analysis with adjusting for relevant confounders.

In our study, rates of baseline and peak ALT derangement between the groups with and without COVID‐19 were significantly different, consistent with findings from a large US cohort.^(^ ^11^ ^)^ The increase in ALT between baseline and at 14 days follow‐up in the group with COVID‐19 is also consistent with previous results.^(^ ^6^ ^)^

Patients with preexisting liver disease had an increased risk of mortality in COVID‐19, which is consistent with the findings of published studies,^(^ ^28^, ^29^ ^)^ although numbers with preexisting liver disease in our study were small. Because some patients with COVID‐19 in our cohort were admitted to hospital for non‐COVID‐19 illnesses, we analyzed baseline liver biochemistry at the time of the SARS‐CoV‐2 RT‐PCR rather than date of admission. This may partially explain differences between our study and previous studies that analyzed liver biochemistry measured on hospital admission.^(^ ^3^, ^4^, ^5^, ^6^ ^)^ Variable patterns of treatment between cohorts may also account for differences.

Although baseline hypoalbuminemia was significantly associated with hazards of death in COVID‐19, ALP and BR were not. Recent findings from two multicenter studies support the prognostic association of albumin with death in COVID‐19,^(^ ^21^, ^30^ ^)^ and a previous prospective study also reported an association of albumin with a composite endpoint (ICU admission or death).^(^ ^17^ ^)^ However, other studies of liver biochemistry in COVID‐19 did not investigate albumin.^(^ ^6^, ^10^, ^11^ ^)^ Interestingly, a previous study found that hypoalbuminemia is a strong predictor of 30‐day all‐cause mortality in acutely admitted medical patients.^(^ ^31^ ^)^ The mechanism of the effect of hypoalbuminemia is not certain; it may reflect the broad association between low albumin and critical illness or be a marker for characteristics of host (e.g., nutritional status, comorbidity) or disease phenotype (e.g., immune activation).^(^ ^32^ ^)^ As albumin is a cheap and widely available test, it can be usefully employed as a prognostic biomarker.

Due to variable population settings in previous studies (e.g., demographics, comorbidities), it is important to understand the populations in which prognostic models are developed^(^ ^15^ ^)^ to ensure such models are externally valid, and to also adjust for relevant confounders. In our cohort, a univariate Cox proportional hazards model and K‐M curve analysis suggest that an elevation in baseline ALT is weakly associated with lower hazards of death from COVID‐19. However, the univariate analysis is unadjusted and may be confounded by additional factors. Interestingly, in a US cohort of 60 hospitalized patients with COVID‐19, ALT at admission was also higher in those who survived compared to those who died.^(^ ^6^ ^)^ In our multivariate analysis, the association of baseline ALT and hazards of death was attenuated toward the null and became nonsignificant after full adjustment for demographics, comorbidities, and prescribed drug use before baseline. Therefore, larger cohorts are warranted to investigate this unexpected association with more confidence. In the sensitivity analysis of patients with COVID‐19 stratified by disease severity, severe/critical cases were more likely to have elevated ALT at baseline compared to mild/moderate cases and were more likely to die compared to mild/moderate cases, in line with other studies.^(^ ^3^, ^12^, ^13^ ^)^

The observed association of raised ALT with lower hazards of death is intriguing as recent data have suggested that genetic predisposition to fatty liver disease, conferred by possession of the patatin‐like phospholipase domain containing 3 [PNPLA3] I148M variant (which is highly prevalent in the UK population),^(^ ^33^ ^)^ is associated with both elevated ALT and a concomitant reduction in systemic inflammation (as measured by CRP) as well as preservation of albumin levels during COVID‐19.^(^ ^34^ ^)^ As such, elevated ALT at baseline may be a surrogate for nonalcoholic fatty liver disease (NAFLD) in a subset of patients; those with NAFLD may have a lower risk of severe COVID‐19 outcomes. However, additional studies are required to confirm this association, and the underlying mechanism is not understood.

A study from the United States^(^ ^11^ ^)^ reported a positive association of ALT >5 times ULN with death; however, we were unable to replicate this investigation as only a small number of participants had ALT elevated to this level. Furthermore, the prevalence of comorbid disease and BMI >35 kg/m^2^ in the US study population was much higher compared to our cohort. Similarly, a large primary care cohort study^(^ ^35^ ^)^ reported that BMI >40 kg/m^2^ was associated with an increased risk of COVID‐19‐related death, but we were underpowered to replicate this analysis.

Although derangements in liver biochemistry are common in patients with COVID‐19, the reasons for the liver injury remain unclear but may include direct viral damage, drug‐induced liver injury, hypoxia, immune‐mediated injury, sepsis, or cytokine release.^(^ ^36^, ^37^ ^)^ Angiotensin‐converting enzyme 2 (ACE2), a functional receptor for SARS‐CoV‐2,^(^ ^38^ ^)^ is found abundantly in the gastrointestinal tract and liver in addition to presenting in alveolar type 2 cells (the major SARS‐CoV‐2‐targeting cell type in lung). A recent study observed a higher expression of ACE2 in cholangiocytes (~60% of cells) compared to hepatocytes (<3%).^(^ ^39^ ^)^ Given the hepatic distribution of the ACE2 receptor, SARS‐CoV‐2 may well cause damage of both bile ducts and liver.^(^ ^40^, ^41^ ^)^ Our stratification analysis by disease severity revealed that compared to patients without COVID‐19, patients with COVID‐19 were more likely to have abnormal liver biochemistry and severe outcomes. Alternatively, the liver may be a bystander, with deranged liver biochemistry reflecting systemic disease.^(^ ^42^ ^)^ It is interesting that liver biochemistry parameters are not currently included in the existing risk stratification tools, such as International Severe Acute Respiratory and Emerging Infection Consortium (ISARIC) scores, for predicting clinical deterioration^(^ ^43^ ^)^ or mortality risk^(^ ^44^ ^)^ for COVID‐19. These parameters (or albumin in particular) may be considered for inclusion in future scoring models or used for early ICU review.

Routinely collected liver biochemistry is not consistent between settings, and therefore the definitions of liver biochemistry derangement may vary across studies. Although AST and GGT have been investigated in previous studies, these parameters were not available for our population. It would be interesting to include GGT in future analysis, and correlation of liver function tests with blood gases would be an approach to determine the extent to which deranged liver biochemistry may be associated with liver hypoxia. Other information, like respiratory parameters, could be included if available in the propensity score matching and multivariate analysis for future analyses. We recognize that analysis can be influenced by missing data, but we have reported missing values and investigated peak/nadir values in addition to baseline. We also undertook sensitivity analysis in a subset of patients with complete BMI measurements to investigate its association with death. Treatment outcomes are important; however, as changing approaches have been made to treatment for COVID‐19 and the drugs used as part of clinical trials were not recorded in the current data set, we have not undertaken an analysis of responses to treatment in this study. Our cohort in the southeast of the United Kingdom may not be representative of populations elsewhere, especially in terms of ethnic diversity, so caution should be applied in extrapolation of results.

Further longitudinal studies of COVID‐19 outcomes in diverse patient groups, including those with preexisting liver disease, are needed. The NIHR HIC program will continue to benefit the field of COVID‐19 research because data accumulated for large teaching hospitals can be used to expand this analysis, resulting in a more generalizable study population and increased statistical power.

In conclusion, liver biochemistry derangement is common in patients with COVID‐19 at the time of a SARS‐CoV‐2 RT‐PCR test and during the clinical course of disease. Baseline hypoalbuminemia and rising ALP over time are prognostic markers for death in patients with COVID‐19, but investigations of larger cohorts are required to develop a better understanding of the relationship between liver biochemistry and disease outcome.

## Supporting information

Supplementary Material
