# Supplementary material for: Longitudinal Analysis of the Utility of Liver Biochemistry as Prognostic Markers in Hospitalized Patients With Corona Virus Disease 2019
Source: Hepatol Commun. 2021 Jul 10;5(9):1586–604. doi: 10.1002/hep4.1739 (PMC8239606; doi:10.1002/hep4.1739)
Supplement: Supplementary file 1 — Supplementary Material [file HEP4-5-1586-s001.docx]

**Supporting tables**

**Supporting Table S1**. **Normal reference and units of blood tests used in this study, based on reference ranges set by the hospital biochemistry lab.**

| Categories | Test name | Normal reference | Units |
| --- | --- | --- | --- |
| Liver function | Alanine transaminase (ALT) | 10-45 | IU/L |
|  | Alkaline phosphatase (ALP) | 30-130 | IU/L |
|  | Bilirubin | 0-21 | umol/L |
|  | Albumin | 32-50 | g/L |
| Blood clotting tests | Prothrombin time (PT) | 9.0-12.0 | seconds |
|  | Activated partial thromboplastin time (APTT) | 20.0-30.0 | seconds |
|  | International normalised ratio (INR) | 0.8-1.2 | ratio |
| Renal function tests | Creatinine | 64-104 | umol/L |
|  | Urea | 3.0-9.2 | mmol/L |
|  | Estimated Glomerular Filtration Rate (eGFR) | ≥90 | ml/min/1.73m^2^ |
| Other tests | C-reactive protein (CRP) | 0-5 | mg/L |
|  | Platelets | 150-400 | x10*9/L |
|  | Lymphocytes | 1.0-4.0 | x10*9/L |

**Supporting Table S2**. **Historical diagnostic ICD codes used to identify pre-existing comorbidities of interest in this study.**

| **Pre-existing comorbidities** | **ICD code categories**  **(retrieved)** | **notes** |
| --- | --- | --- |
| Liver disease | B18 category | Chronic viral hepatitis |
|  | K70-K77 categories | Diseases of liver   - K70 category: Alcoholic liver disease - K71 category: Toxic liver disease - K72 category: Hepatic failure, not elsewhere classified - K73 category: Chronic hepatitis, not elsewhere classified - K74 category: Fibrosis and cirrhosis of liver - K75 category: Other inflammatory liver diseases - K76 category: Other diseases of liver - K77 category: Liver disorders in diseases classified elsewhere |
| DM | E10-E14 categories | Diabetes mellitus |
| HTN | I10 category | Essential (primary) hypertension |
| CHD | I20-I25 categories | Coronary heart disease, *also called* ischaemic heart disease |
| CKD | N18 category | Chronic kidney disease |
| Cancer | C00-C97 categories | Malignant neoplasms |

*CHD, Coronary heart disease; CKD, Chronic kidney disease; DM, Diabetes mellitus; HTN, Hypertension.*

**Supporting Table S3**. **Detailed information for the full list of drugs searched for in this cohort, and drugs used in the study cohort**.

| **Type of drugs** | **The full drug list searched**^§^ | **Drugs used in the study cohort**^#^ |
| --- | --- | --- |
| Antiviral drugs | Abacavir\|Aciclovir\|Acyclovir\|Adefovir\|Amantadine\|Ampligen\|Agenerase\|Amprenavir\|ArbidolUmifenovir\|Atazanavir\|Atripla\|Xofluza\|Baloxavir marboxil\|Biktarvy\|Boceprevir\|Cidofovir\|Tybost\|Cobicistat\|Combivir\|Daklinza\|Daclatasvir\|Darunavir\|Delavirdine\|Descovy\|Didanosine\|Docosanol\|Dolutegravir\|Pifeltro\|Doravirine\|Edoxudine\|Efavirenz\|Elvitegravir\|Emtricitabine\|Enfuvirtide\|Entecavir\|Intelence\|Etravirine\|Famciclovir\|Fomivirsen\|Fosamprenavir\|Foscarnet\|Cytovene\|Ganciclovir\|Ibacitabine\|Trogarzo\|Ibalizumab\|Idoxuridine\|Imiquimod\|Imunovir\|Indinavir\|Lamivudine\|Prevymis\|Letermovir\|Lopinavir\|Loviride\|Maraviroc\|Methisazone\|Moroxydine\|Nelfinavir\|Nevirapine\|Kutapressin\|Nexavir formerly\|Nitazoxanide\|Norvir\|Oseltamivir\|Penciclovir\|Peramivir\|Penciclovir\|Rapivab\|Peramivir\|Pleconaril\|Podophyllotoxin\|Raltegravir\|Remdesivir\|Ribavirin\|Edurant\|Rilpivirine\|Rilpivirine\|Rimantadine\|Ritonavir\|Saquinavir\|Olysio\|Simeprevir\|Sofosbuvir\|Stavudine\|Viramidine\|Taribavirin\|Telaprevir\|Tyzeka\|Telbivudine\|Tenofovir alafenamide\|Tenofovir disoproxil\|Tenofovir\|Tipranavir\|Trifluridine\|Trizivir\|Tromantadine\|Truvada\|Umifenovir\|Valaciclovir\|Valtrex\|Valganciclovir\|Vicriviroc\|Vidarabine\|Zalcitabine\|Relenza\|Zanamivir\|Zidovudine | Abacavir  Aciclovir  Amantadine  Cidofovir  Darunavir  Dolutegravir  Efavirenz  Emtricitabine  Entecavir  Imiquimod  Lamivudine  Letermovir  Lopinavir  Oseltamivir  Raltegravir  Remdesivir  Ritonavir  Tenofovir  Tenofovir alafenamide  Tenofovir disoproxil  Valganciclovir  Zanamivir |
| Antibiotics | Amoxicillin\|amoxiclav\|CefTRIAXone\|penicillin\|Piperacillin\|Flucloxacillin\|claRITHromycin\|Vancomycin | Amoxicillin  Benzylpenicillin Sodium  Ceftriaxone  Clarithromycin  Co-Amoxiclav  Flucloxacillin  Phenoxymethylpenicillin  Piperacillin  Vancomycin |
| Anticoagulants | warfarin\|enoxaparin\|Lovenox\|dalteparin\|Fragmin\|heparinbivalirudin\|Angiomax\|argatroban\|Acova\|dabigatran\|Pradaxa\|antithrombin\|Thrombate\|apixaban\|Eliquis\|fondaparinux\|Arixtra\|rivaroxaban\|Xarelto\|edoxaban\|Savaysa | Apixaban  Dabigatran etexilate  Dalteparin  Edoxaban  Enoxaparin  Fondaparinux  Rivaroxaban  Warfarin |
| Acetaminophen | Acetaminophen\|Paracetamol | Acetaminophen (Paracetamol) |
| Immunosuppressants | Azathioprine\|Cyclophosphamide\|Cyclosporine\|Methotrexate\|Mycophenolate mofetil\| Mycophenolic acid | Azathioprine  Cyclophosphamide  Methotrexate  Mycophenolate mofetil  Mycophenolic acid |
| Statins | regex('statin', ignore_case = T)^‡^ | Atorvastatin  Fluvastatin  Pravastatin  Rosuvastatin  Simvastatin |

^§^ Regular expressions were used to retrieve the drug information. ^‡^Statins were searched using a regular expression including 'statin'. ^#^The drugs used as part of clinical trials were not recorded in the dataset.

**Supporting Table S4. Demographics and comorbidity characteristics of patients with and without COVID-19 before and after propensity score matching.**

|  | Before matching | | | After matching | | |
| --- | --- | --- | --- | --- | --- | --- |
| Variables  used for matching | COVID-19 Positive  (n=585) | COVID-19 Negative  (n=5726) | p-value | COVID-19 Positive  (n=585) | COVID-19 Negative  (n=1165) | p-value |
| Gender = male (%) | 312 (53.3) | 2901 (50.7) | 0.235 | 312 (53.3) | 629 (54.0) | 0.834 |
| Age at test (median [IQR]) | 73 [57, 84] | 68 [53, 81] | **<0.001** | 73 [57, 84] | 73 [58, 83] | 0.755 |
| Ethnicity category (%) |  |  | **<0.001** |  |  | 0.911 |
| Asian | 31 (5.3) | 166 (2.9) |  | 31 (5.3) | 62 (5.3) |  |
| Black | 20 (3.4) | 65 (1.1) |  | 20 (3.4) | 35 (3.0) |  |
| Mixed | 10 (1.7) | 41 (0.7) |  | 10 (1.7) | 22 (1.9) |  |
| Not stated | 100 (17.1) | 1062 (18.5) |  | 100 (17.1) | 219 (18.8) |  |
| Other | 9 (1.5) | 43 (0.8) |  | 9 (1.5) | 23 (2.0) |  |
| White | 415 (70.9) | 4349 (76.0) |  | 415 (70.9) | 804 (69.0) |  |
| **Pre-existing**  **comorbidities,**  **n (%)** |  |  |  |  |  |  |
| Liver disease | 17 (2.9) | 210 (3.7) | 0.409 | 17 (2.9) | 33 (2.8) | 1 |
| DM | 88 (15.0) | 572 (10.0) | **<0.001** | 88 (15.0) | 175 (15.0) | 1 |
| HTN | 179 (30.6) | 1100 (19.2) | **<0.001** | 179 (30.6) | 349 (30.0) | 0.825 |
| CHD | 59 (10.1) | 441 (7.7) | 0.051 | 59 (10.1) | 113 (9.7) | 0.864 |
| CKD | 56 (9.6) | 400 (7.0) | **0.027** | 56 (9.6) | 107 (9.2) | 0.86 |
| Cancer | 41 (7.0) | 628 (11.0) | **0.004** | 41 (7.0) | 88 (7.6) | 0.753 |

**Supporting Table S5. Distribution of specialities under which patients in COVID-19 and non-COVID-19 groups were admitted to hospital.**

|  | **COVID-19 group**  **(n=585)** | | **non-COVID-19 group**  **(n=1165)** | |
| --- | --- | --- | --- | --- |
| **Specialty** | **count** | **percentage** | **count** | **percentage** |
| General Internal Medicine | 213 | 36.40% | 267 | 22.90% |
| Geriatric Medicine | 115 | 19.70% | 87 | 7.50% |
| Infectious Diseases | 25 | 4.30% | 48 | 4.10% |
| Respiratory Medicine | 23 | 3.90% | 25 | 2.10% |
| Emergency Medicine | 22 | 3.80% | 44 | 3.80% |
| Gastroenterology | 21 | 3.60% | 42 | 3.60% |
| General Surgery | 19 | 3.20% | 213 | 18.30% |
| Trauma and Orthopaedics | 15 | 2.60% | 77 | 6.60% |
| Renal Medicine | 13 | 2.20% | 28 | 2.40% |
| Endocrinology and Diabetes | 12 | 2.10% | 3 | 0.30% |
| Clinical Haematology | 10 | 1.70% | 21 | 1.80% |
| Cardiology | 8 | 1.40% | 43 | 3.70% |
| Clinical Pharmacology | 4 | 0.70% | 2 | 0.20% |
| Medical Oncology | 4 | 0.70% | 10 | 0.90% |
| Urology | 3 | 0.50% | 32 | 2.70% |
| Neurosurgery | 3 | 0.50% | 26 | 2.20% |
| Cardiothoracic Surgery | 3 | 0.50% | 12 | 1.00% |
| Plastic Surgery | 2 | 0.30% | 4 | 0.30% |
| Neurology | 2 | 0.30% | 9 | 0.80% |
| Gynaecology | 2 | 0.30% | 13 | 1.10% |
| Ophthalmology | 1 | 0.20% | 4 | 0.30% |
| Oral and Maxillofacial Surgery | 1 | 0.20% | 4 | 0.30% |
| Intensive Care Medicine | 1 | 0.20% | 7 | 0.60% |
| Palliative Medicine | 1 | 0.20% | 7 | 0.60% |
| Rheumatology | 1 | 0.20% | 2 | 0.20% |
| Clinical Oncology | 1 | 0.20% | 7 | 0.60% |
| Ear Nose and Throat |  |  | 5 | 0.40% |
| Radiology |  |  | 5 | 0.40% |
| Rehabilitation Medicine |  |  | 3 | 0.30% |
| Anaesthetics |  |  | 1 | 0.10% |
| Dermatology |  |  | 1 | 0.10% |
| Medical Microbiology |  |  | 1 | 0.10% |
| Midwifery |  |  | 1 | 0.10% |
| Obstetrics |  |  | 1 | 0.10% |
| Unknown | 60 | 10.30% | 110 | 9.40% |

**Supporting Table S6. Comparison of baseline and peak/nadir liver biochemistry in patients with and without COVID-19, stratified by various degrees of derangement**.

|  | **Baseline liver biochemistry**^§^ | | | **Peak/nadir liver biochemistry**^†^ | | |
| --- | --- | --- | --- | --- | --- | --- |
|  | COVID-19  group  n=492 | Non-COVID-19 group  n=974 | p-  value | COVID-19  group  n=585 | non-COVID-19 group  n=1165 | p-  value |
| **Number of**  **deranged liver biochemistry, (%)** |  |  |  |  |  |  |
| 0 | 135 (27.4) | 433 (44.5) | **<0.001** | 80 (13.7) | 323 (27.7) | **<0.001** |
| 1 | 234 (47.6) | 337 (34.6) | **<0.001** | 212 (36.2) | 350 (30.0) | **0.01** |
| 2 | 92 (18.7) | 146 (15.0) | 0.081 | 169 (28.9) | 298 (25.6) | 0.16 |
| 3 | 25 (5.1) | 42 (4.3) | 0.59 | 92 (15.7) | 123 (10.6) | **0.002** |
| 4 | 6 (1.2) | 16 (1.6) | 0.69 | 32 (5.5) | 71 (6.1) | 0.68 |
| ALT (median [IQR]), IU/L | 25 [16, 40] | 19 [13, 32] | **<0.001** | 34 [20, 66] | 26 [17, 51] | **<0.001** |
| ALT, >ULN, n(%) | 102 (20.7) | 142 (14.6) | **0.004** | 222 (37.9) | 323 (27.7) | **<0.001** |
| **ALT (categories), n(%)** |  |  |  |  |  |  |
| normal | 390 (79.3) | 832 (85.4) | **0.004** | 363 (62.1) | 842 (72.3) | **<0.001** |
| >1-2ULN | 64 (13.0) | 88 (9.0) | **0.023** | 115 (19.7) | 184 (15.8) | 0.050 |
| >2-3ULN | 21 (4.3) | 20 (2.1) | **0.024** | 39 (6.7) | 49 (4.2) | **0.035** |
| >3ULN | 17 (3.5) | 34 (3.5) | 1 | 68 (11.6) | 90 (7.7) | **0.009** |
| ALP (median [IQR]), IU/L | 85 [66, 114] | 90 [71, 124] | **0.005** | 106 [79, 155] | 104 [78, 149] | 0.69 |
| ALP, >ULN, n(%) | 98 (19.8) | 213 (21.6) | 0.47 | 199 (34.0) | 382 (32.8) | 0.65 |
| **ALP (abnormal**  **categories), n(%)** |  |  |  |  |  |  |
| normal | 396 (80.2) | 772 (78.4) | 0.47 | 386 (66.0) | 783 (67.2) | 0.65 |
| >1-2ULN | 82 (16.6) | 163 (16.5) | 1 | 146 (25.0) | 278 (23.9) | 0.66 |
| >2-3ULN | 9 (1.8) | 28 (2.8) | 0.31 | 32 (5.5) | 55 (4.7) | 0.57 |
| >3ULN | 7 (1.4) | 22 (2.2) | 0.39 | 21 (3.6) | 49 (4.2) | 0.62 |
| Bilirubin (median [IQR]), umol/L | 9 [6, 13] | 10 [7, 16] | **<0.001** | 11 [8, 16] | 12 [8, 17] | 0.20 |
| Bilirubin, >ULN, n(%) | 29 (5.9) | 127 (13.0) | **<0.001** | 71 (12.1) | 201 (17.3) | **0.007** |
| **Bilirubin (categories), n(%)** |  |  |  |  |  |  |
| normal | 463 (94.1) | 847 (87.0) | **<0.001** | 514 (87.9) | 964 (82.7) | **0.007** |
| >1-2ULN | 22 (4.5) | 89 (9.1) | **0.002** | 52 (8.9) | 135 (11.6) | 0.10 |
| >2-3ULN | 3 (0.6) | 17 (1.7) | 0.13 | 9 (1.5) | 29 (2.5) | 0.27 |
| >3ULN | 4 (0.8) | 21 (2.2) | 0.097 | 10 (1.7) | 37 (3.2) | 0.10 |
| Albumin (median [IQR]), g/L | 30 [27, 34] | 34 [29, 37] | **<0.001** | 26 [21, 31] | 29 [24, 35] | **<0.001** |
| Albumin, <LLN, n(%) | 291 (58.7) | 346 (35.0) | **<0.001** | 462 (79.0) | 693 (59.5) | **<0.001** |

^§^ Only 492 vs. 974 patients in COVID-19 group vs. non-COVID-19 group had data of all the liver biochemistry available at baseline. ^†^ For ALT, ALP, bilirubin, we investigated the peak values while nadir values for albumin. For categorical variables, Fisher exact test was performed for comparison on cells with small counts (<5), otherwise Chi-square test was used. For continuous variables, Wilcoxon test was used for comparison due to non-normality. *ALT, Alanine transaminase; ALP, Alkaline phosphatase;LLN, Lower limit of normal; ULN, Upper limit of normal.*

**Supporting Table S7**. **Comparison of demographics, comorbidities, baseline and peak/nadir liver biochemistry, and outcomes between Mild/Moderate COVID-19 cases and non-COVID-19 patients.**

|  | **Mild/Moderate COVID-19 cases**  **(n=358)** ^†^ | **non-COVID-19 patients**  **(n=838)** | p-value |
| --- | --- | --- | --- |
| Gender = male (%) | 198 (55.3) | 460 (54.9) | 0.945 |
| Age at test (median [IQR]) | 74.0 [59.0, 83.8] | 76.0 [60.0, 85.0] | 0.265 |
| **Ethnicity category (%)** |  |  | 0.858 |
| Asian | 19 (5.3) | 36 (4.3) |  |
| Black | 11 (3.1) | 19 (2.3) |  |
| Mixed | 5 (1.4) | 15 (1.8) |  |
| White | 260 (72.6) | 608 (72.6) |  |
| Other | 4 (1.1) | 13 (1.6) |  |
| Not stated | 59 (16.5) | 147 (17.5) |  |
| BMI(median [IQR]), kg/m^2^ | 26.9 [23.6, 30.9] | 26.0 [22.6, 30.6] | 0.126 |
| BMI category, n(%) |  |  | 0.255 |
| <18.5 | 9 (3.4) | 31 (5.1) |  |
| ≥18.5 - <25 | 90 (34.0) | 235 (38.5) |  |
| ≥25 - <30 | 85 (32.1) | 172 (28.2) |  |
| ≥30 - <35 | 52 (19.6) | 91 (14.9) |  |
| ≥35 - <40 | 18 (6.8) | 52 (8.5) |  |
| ≥40 | 11 (4.2) | 30 (4.9) |  |
| **Pre-existing comorbidities, n(%)** |  |  |  |
| Liver disease | 10 (2.8) | 27 (3.2) | 0.834 |
| DM | 52 (14.5) | 147 (17.5) | 0.231 |
| HTN | 109 (30.4) | 275 (32.8) | 0.462 |
| CHD | 36 (10.1) | 90 (10.7) | 0.803 |
| CKD | 33 (9.2) | 78 (9.3) | 1 |
| Cancer | 27 (7.5) | 65 (7.8) | 0.993 |
| **Baseline liver biochemistry** |  |  |  |
| ALT, >ULN, n(%) | 66 (19.0) | 111 (13.7) | **0.026** |
| ALT (median [IQR]), IU/L | 24.0 [17.0, 39.0] | 19.0 [13.0, 31.0] | **<0.001** |
| ALP, >ULN, n(%) | 71 (20.4) | 185 (22.7) | 0.425 |
| ALP (median [IQR]), IU/L | 84.0 [66.0, 116.2] | 90.0 [72.0, 126.0] | **0.002** |
| Bilirubin, >ULN, n(%) | 21 (6.1) | 102 (12.6) | **0.001** |
| Bilirubin (median [IQR]), umol/L | 9.0 [6.0, 14.0] | 10.0 [7.0, 16.0] | **0.002** |
| Albumin, <LLN, n(%) | 201 (57.8) | 291 (35.7) | **<0.001** |
| Albumin (median [IQR]), g/L | 30.5 [27.0, 34.0] | 34.0 [29.0, 37.0] | **<0.001** |
| **Peak/Nadir liver biochemistry** |  |  |  |
| ALT, >ULN, n(%) | 148 (41.3) | 243 (29.0) | **<0.001** |
| ALT (median [IQR]), IU/L | 37.0 [23.0, 65.8] | 28.0 [17.0, 52.0] | **<0.001** |
| ALP, >ULN, n(%) | 133 (37.2) | 302 (36.0) | 0.764 |
| ALP (median [IQR]), IU/L | 108.0 [81.0, 158.0] | 107.5 [81.2, 159.8] | 0.817 |
| Bilirubin, >ULN, n(%) | 52 (14.5) | 153 (18.3) | 0.138 |
| Bilirubin (median [IQR]), umol/L | 12.0 [8.0, 17.0] | 12.0 [8.0, 18.0] | 0.333 |
| Albumin, <LLN, n(%) | 307 (85.8) | 543 (64.8) | **<0.001** |
| Albumin (median [IQR]), g/L | 25.0 [21.0, 29.0] | 29.0 [24.0, 33.0] | **<0.001** |
| **Outcomes** |  |  |  |
| Death, n(%) | 97 (27.1) | 114 (13.6) | **<0.001** |
| ICU admission, n(%) | 37 (10.3) | 26 (3.1) | **<0.001** |
| Used invasive ventilation in ICU, n(%) | 23 (6.4) | 13 (1.6) | **<0.001** |

^†^ Mild/Moderate: baseline respiratory rate ≤30 breaths/min and oxygen saturation (SpO2) ≥90%. ^§^ 327 non-COVID-19 patients missing data on respiratory rate and oxygen saturation. For ALT, ALP, bilirubin, we investigated the peak values while nadir values for albumin. For categorical variables, Fisher exact test was performed for comparison on cells with small counts (<5), otherwise Chi-square test was used. For continuous variables, Wilcoxon test was used for comparison due to non-normality. *ALT, Alanine transaminase; ALP, Alkaline phosphatase; LLN, Lower limit of normal; ULN, Upper limit of normal.*

**Supporting Table S8**. **Comparison of demographics, comorbidities, baseline and peak/nadir liver biochemistry, and outcomes between Severe/Critical COVID-19 cases and non-COVID-19 patients.**

|  | **Severe/Critical COVID-19 cases**  **(n=65)** ^‡^ | **non-COVID-19 patients**  **(n=838)^§^** | p-value |
| --- | --- | --- | --- |
| Gender = male (%) | 31 (47.7) | 460 (54.9) | 0.320 |
| Age at test (median [IQR]) | 71.0 [56.0, 84.0] | 76.0 [60.0, 85.0] | 0.295 |
| **Ethnicity category (%)** |  |  | 0.567 |
| Asian | 1 (1.5) | 36 (4.3) |  |
| Black | 2 (3.1) | 19 (2.3) |  |
| Mixed | 3 (4.6) | 15 (1.8) |  |
| White | 48 (73.8) | 608 (72.6) |  |
| Other | 1 (1.5) | 13 (1.6) |  |
| Not stated | 10 (15.4) | 147 (17.5) |  |
| BMI(median [IQR]), kg/m^2^ | 25.7 [21.6, 32.0] | 26.0 [22.6, 30.6] | 0.937 |
| BMI category, n(%) |  |  | 0.357 |
| <18.5 | 2 (5.4) | 31 (5.1) |  |
| ≥18.5 - <25 | 15 (40.5) | 235 (38.5) |  |
| ≥25 - <30 | 6 (16.2) | 172 (28.2) |  |
| ≥30 - <35 | 10 (27.0) | 91 (14.9) |  |
| ≥35 - <40 | 3 (8.1) | 52 (8.5) |  |
| ≥40 | 1 (2.7) | 30 (4.9) |  |
| **Pre-existing comorbidities, n(%)** |  |  |  |
| Liver disease | 2 (3.1) | 27 (3.2) | 1 |
| DM | 7 (10.8) | 147 (17.5) | 0.220 |
| HTN | 13 (20.0) | 275 (32.8) | **0.046** |
| CHD | 5 (7.7) | 90 (10.7) | 0.574 |
| CKD | 3 (4.6) | 78 (9.3) | 0.294 |
| Cancer | 3 (4.6) | 65 (7.8) | 0.496 |
| **Baseline liver biochemistry** |  |  |  |
| ALT, >ULN, n(%) | 20 (31.2) | 111 (13.7) | **<0.001** |
| ALT (median [IQR]), IU/L | 29.5 [18.8, 50.2] | 19.0 [13.0, 31.0] | **<0.001** |
| ALP, >ULN, n(%) | 10 (15.6) | 185 (22.7) | 0.246 |
| ALP (median [IQR]), IU/L | 80.0 [60.8, 104.0] | 90.0 [72.0, 126.0] | **0.005** |
| Bilirubin, >ULN, n(%) | 2 (3.1) | 102 (12.6) | **0.04** |
| Bilirubin (median [IQR]), umol/L | 10.0 [7.0, 13.2] | 10.0 [7.0, 16.0] | 0.445 |
| Albumin, <LLN, n(%) | 39 (60.9) | 291 (35.7) | **<0.001** |
| Albumin (median [IQR]), g/L | 30.5 [26.8, 33.2] | 34.0 [29.0, 37.0] | **<0.001** |
| **Peak/Nadir liver biochemistry** |  |  |  |
| ALT, >ULN, n(%) | 29 (44.6) | 243 (29.0) | **0.012** |
| ALT (median [IQR]), IU/L | 39 [26, 97] | 28 [17, 52] | **0.001** |
| ALP, >ULN, n(%) | 18 (27.7) | 302 (36.0) | 0.222 |
| ALP (median [IQR]), IU/L | 95.0 [75.0, 152.0] | 107.5 [81.2, 159.8] | 0.105 |
| Bilirubin, >ULN, n(%) | 5 (7.7) | 153 (18.3) | **0.047** |
| Bilirubin (median [IQR]), umol/L | 13.0 [9.0, 16.0] | 12.0 [8.0, 18.0] | 0.988 |
| Albumin, <LLN, n(%) | 57 (87.7) | 543 (64.8) | **<0.001** |
| Albumin (median [IQR]), g/L | 25.0 [21.0, 28.0] | 29.0 [24.0, 33.0] | **<0.001** |
| **Outcomes** |  |  |  |
| Death, n(%) | 28 (43.1) | 114 (13.6) | **<0.001** |
| ICU admission, n(%) | 18 (27.7) | 26 (3.1) | **<0.001** |
| Used invasive ventilation in ICU, n(%) | 14 (21.5) | 13 (1.6) | **<0.001** |

^‡^ Severe/Critical: respiratory rate >30 breaths/min, or oxygen saturation(SpO2) <90%. ^§^ 327 non-COVID-19 patients missing data on respiratory rate and oxygen saturation. For ALT, ALP, bilirubin, we investigated the peak values while nadir values for albumin. For categorical variables, Fisher exact test was performed for comparison on cells with small counts (<5), otherwise Chi-square test was used. For continuous variables, Wilcoxon test was used for comparison due to non-normality. *ALT, Alanine transaminase; ALP, Alkaline phosphatase; LLN, Lower limit of normal; ULN, Upper limit of normal.*

**Supporting Table S9**. **Characteristics of baseline and peak of ALP and bilirubin of patients, stratified by death status in COVID-19 group**.

|  | **Survived (n=428)** | **Died (n=157)** | **p-value** |
| --- | --- | --- | --- |
| Baseline ALP (median [IQR]), IU/L | 81 [64, 110] | 89 [73, 122] | **0.007** |
| **Baseline ALP categories^†^, n(%)** |  |  |  |
| Normal | 284 (81.6) | 112 (76.7) | 0.26 |
| >1-2ULN | 55 (15.8) | 27 (18.5) | 0.55 |
| >2-3ULN | 4 (1.1) | 5 (3.4) | 0.18 |
| >3ULN | 5 (1.4) | 2 (1.4) | 1 |
| Baseline bilirubin (median [IQR]), umol/L | 9 [6, 13] | 10 [6, 15] | 0.056 |
| **Baseline bilirubin categories^‡^, n(%)** |  |  |  |
| Normal | 329 (95.1) | 134 (91.8) | 0.23 |
| >1-2ULN | 13 (3.8) | 9 (6.2) | 0.35 |
| >2-3ULN | 2 (0.6) | 1 (0.7) | 1 |
| >3ULN | 2 (0.6) | 2 (1.4) | 0.73 |
| Peak ALP (median [IQR]), IU/L | 105 [77, 151] | 110 [84, 179] | 0.076 |
| **Peak ALP categories, n(%)** |  |  |  |
| Normal | 290 (67.8) | 96 (61.1) | 0.16 |
| >1-2ULN | 104 (24.3) | 42 (26.8) | **0.001** |
| >2-3ULN | 15 (3.5) | 17 (10.8) | 0.91 |
| >3ULN | 19 (4.4) | 2 (1.3) | **<0.001** |
| Peak bilirubin (median [IQR]), umol/L | 11 [8, 15] | 12 [8, 18] | 0.096 |
| **Peak bilirubin categories, n(%)** |  |  |  |
| Normal | 385 (90.0) | 129 (82.2) | **0.016** |
| >1-2ULN | 32 (7.5) | 20 (12.7) | 0.069 |
| >2-3ULN | 5 (1.2) | 4 (2.5) | 0.41 |
| >3ULN | 6 (1.4) | 4 (2.5) | 0.56 |

^†^ 80 vs. 11 patients in alive subgroup vs. died subgroup had no data of ALP available at baseline. ^‡^ 82 vs. 11 patients in alive subgroup vs. died subgroup had no data of bilirubin available at baseline. For categorical variables, Fisher exact test was performed for comparison on cells with small counts (<5), otherwise Chi-square test was used. For continuous variables, Wilcoxon test was used for comparison due to non-normality. *ALP, Alkaline phosphatase; IQR, Interquartile range; ULN, Upper limit of normal.*

**Supporting Table S10. Patient characteristics, demographics, liver biochemistry, and clinical information in non-COVID-19 group, stratified by survival status**.

|  | **Survived (n=1026)** | **Died (n=139)** | **p-value** |
| --- | --- | --- | --- |
| Gender = male (%) | 545 (53.1) | 84 (60.4) | 0.125 |
| Age at test (median [IQR]) | 72 [56, 83] | 81 [71, 87] | **<0.001** |
| Age ≥ 75 years (%) | 450 (43.9) | 92 (66.2) | **<0.001** |
| **Ethnicity category (%)** |  |  |  |
| Asian | 59 (5.8) | 3 (2.2) | 0.12 |
| Black | 33 (3.2) | 2 (1.4) | 0.38 |
| Mixed and other | 41 (3.9) | 4 (2.9) | 0.68 |
| White | 695 (67.7) | 109 (78.4) | **0.014** |
| Not stated | 198 (19.3) | 21 (15.1) | 0.28 |
| Used invasive  ventilation in ICU, n(%) | 22 (2.1) | 7 (5.0) | 0.078 |
| ICU admission, n(%) | 36 (3.5) | 14 (10.1) | **0.001** |
| **Pre-existing comorbidities, n(%)** |  |  |  |
| Liver disease | 28 (2.7) | 5 (3.6) | 0.759 |
| DM | 144 (14.0) | 31 (22.3) | **0.015** |
| HTN | 294 (28.7) | 55 (39.6) | **0.011** |
| CHD | 94 (9.2) | 19 (13.7) | 0.125 |
| CKD | 88 (8.6) | 19 (13.7) | 0.073 |
| Cancer | 64 (6.2) | 24 (17.3) | **<0.001** |
| ≥1 liver biochemistry abnormal at baseline^†^, n (%) | 442 (52.4) | 99 (76.2) | **<0.001** |
| ≥1 liver biochemistry abnormal  at baseline (excl. albumin) ^‡^, n (%) | 284 (33.5) | 70 (53.8) | **<0.001** |
| Baseline ALT (median [IQR]) | 19.0 [13.0, 32.0] | 20.5 [12.0, 33.5] | 0.926 |
| **Baseline ALT categories**^§^**, n(%)** |  |  |  |
| normal | 721 (85.4) | 111 (85.4) | 1 |
| >1-2ULN | 80 (9.5) | 8 (6.2) | 0.29 |
| >2-3ULN | 18 (2.1) | 2 (1.5) | 0.91 |
| >3ULN | 25 (3.0) | 9 (6.9) | **0.042** |
| Baseline ALP (median [IQR]) | 88.0 [70.0, 119.0] | 110.0 [81.0, 170.5] | **<0.001** |
| **Baseline ALP categories**^¶^**, n(%)** |  |  |  |
| Normal | 694 (81.2) | 78 (60.0) | **<0.001** |
| >1-2ULN | 125 (14.6) | 38 (29.2) | **<0.001** |
| >2-3ULN | 22 (2.6) | 6 (4.6) | 0.31 |
| >3ULN | 14 (1.6) | 8 (6.2) | **0.003** |
| Baseline bilirubin (median [IQR]), umol/L | 10.0 [7.0, 15.0] | 10.5 [7.0, 17.0] | 0.32 |
| **Baseline bilirubin categories**^#^**, n(%)** |  |  |  |
| Normal | 739 (87.6) | 108 (83.1) | 0.20 |
| >1-2ULN | 75 (8.9) | 14 (10.8) | 0.59 |
| >2-3ULN | 13 (1.5) | 4 (3.1) | 0.38 |
| >3ULN | 17 (2.0) | 4 (3.1) | 0.65 |
| Baseline albumin (median [IQR]), g/L | 34.0 [30.0, 38.0] | 30.5 [26.0, 34.0] | **<0.001** |
| Baseline albumin (<LLN)^*^, n(%) | 273 (31.8) | 73 (56.2) | **<0.001** |
| ≥1 peak/nadir liver biochemistry  abnormal, n(%) | 711 (69.3) | 131 (94.2) | **<0.001** |
| ≥1 peak liver biochemistry  abnormal (excl. albumin), n (%) | 496 (48.3) | 102 (73.4) | **<0.001** |
| Peak ALT (median [IQR]), IU/L | 26.0 [17.0, 47.0] | 35.0 [17.5, 93.0] | **0.002** |
| **Peak ALT categories, n(%)** |  |  |  |
| normal | 757 (73.8) | 85 (61.2) | **0.003** |
| >1-2ULN | 165 (16.1) | 19 (13.7) | 0.54 |
| >2-3ULN | 41 (4.0) | 8 (5.8) | 0.46 |
| >3ULN | 63 (6.1) | 27 (19.4) | **<0.001** |
| Peak ALP (median [IQR]), IU/L | 101.0 [76.0, 141.0] | 146.0 [103.5, 222.5] | **<0.001** |
| **Peak ALP categories (%)** |  |  |  |
| Normal | 726 (70.8) | 57 (41.0) | **<0.001** |
| >1-2ULN | 226 (22.0) | 52 (37.4) | **<0.001** |
| >2-3ULN | 37 (3.6) | 18 (12.9) | **<0.001** |
| >3ULN | 37 (3.6) | 12 (8.6) | **0.011** |
| Peak bilirubin (median [IQR]), umol/L | 11.0 [8.0, 17.0] | 13.0 [8.0, 24.0] | **0.009** |
| **Peak bilirubin categories (%)** |  |  |  |
| Normal | 864 (84.2) | 100 (71.9) | **0.001** |
| >1-2ULN | 114 (11.1) | 21 (15.1) | 0.22 |
| >2-3ULN | 23 (2.2) | 6 (4.3) | 0.24 |
| >3ULN | 25 (2.4) | 12 (8.6) | **<0.001** |
| Nadir albumin (median [IQR]), g/L | 30.0 [25.0, 35.0] | 23.0 [19.0, 27.0] | **<0.001** |
| Nadir albumin (<LLN), n(%) | 568 (55.4) | 125 (89.9) | **<0.001** |

^†^ 182 vs. 9 patients in survived subgroup vs. died subgroup had not all liver biochemistry baseline data available. ^‡^ 179 vs. 9 patients in survived subgroup vs. died subgroup had not all liver biochemistry (excl. albumin) baseline data available at baseline. ^§^ 182 vs. 9 patients in survived subgroup vs. died subgroup had no data of ALT available at baseline. ^¶^ 171 vs. 9 patients in survived subgroup vs. died subgroup had no data of ALP available at baseline. ^#^ 182 vs. 9 patients in survived subgroup vs. died subgroup had no data of bilirubin available at baseline. ^*^ 168 vs. 9 patients in survived subgroup vs. died subgroup had no data of albumin available at baseline. NB. For categorical variables, Fisher exact test was performed for comparison on cells with small counts (<5), otherwise Chi-square test was used. For continuous variables, Wilcoxon test was used for comparison due to non-normality. *ALT, Alanine transaminase; ALP, Alkaline phosphatase; CHD, Coronary heart disease; CKD, Chronic kidney disease; DM, Diabetes mellitus; HTN, Hypertension; IQR, Interquartile range; LLN, Lower limit of normal; ULN, Upper limit of normal.*

**Supporting Table S11. Associations of baseline liver biochemistries with death among patients with COVID-19, investigated with Cox proportional-hazards modelling**.

|  | Univariate analysis | | Multivariate analysis | |
| --- | --- | --- | --- | --- |
| Variables | Crude  HR (95% CIs) | p-value | Adjusted  HR (95% CIs) | p-value |
| Age (divided by 10 years) | 1.74 (1.53-1.97) | **<0.001** | **1.82 (1.56-2.13)** | **<0.001** |
| Gender (male) | 1.24 (0.91-1.71) | 0.18 | 1.25 (0.88-1.76) | 0.21 |
| Ethnicity (white) | 1.87 (1.25-2.8) | **0.002** | 1.09 (0.69-1.72) | 0.71 |
| Baseline ALT (IU/L) | 1 (0.99-1) | 0.39 |  |  |
| Baseline ALP (IU/L) | 1 (1-1) | 0.35 |  |  |
| Baseline bilirubin (umol/L) | 1.01 (1-1.02) | 0.18 |  |  |
| (- Baseline albumin) ^†^ (g/L) | 1.06 (1.04-1.09) | **<0.001** | **1.05 (1.02-1.09)** | **0.002** |
| Pre-existing Liver disease | 2.66 (1.44-4.9) | **0.002** | **3.15 (1.47-6.74)** | **0.003** |
| Pre-existing DM | 1.7 (1.16-2.48) | **0.006** | 1.43 (0.91-2.24) | 0.125 |
| Pre-existing HTN | 1.33 (0.96-1.84) | 0.085 | 0.66 (0.41-1.04) | 0.075 |
| Pre-existing CHD | 1.72 (1.11-2.65) | **0.015** | 1.02 (0.59-1.78) | 0.93 |
| Pre-existing CKD | 1.34 (0.83-2.17) | 0.23 | 0.76 (0.42-1.38) | 0.37 |
| Pre-existing Cancer | 1.71 (1.04-2.84) | **0.036** | 0.89 (0.48-1.65) | 0.71 |

^†^ To make it more interpretable, we transfer the baseline albumin values to negative values. *NB*. Analyses were performed on the 492 COVID-19 patients with baseline liver biochemistry data available (survived vs. died: 346 vs. 146); variables (except for demographics, comorbidities) with p<0.1 in univariate analysis were included for multivariate analysis; in the multivariate analysis, HRs were fully adjusted for drugs use before baseline (including antiviral drugs, antibiotics, anticoagulants, acetaminophen, immunosuppressants, statins) to reduce confounding effects. *ALT, Alanine transaminase; ALP, Alkaline phosphatase; CHD, Coronary heart disease; CKD, Chronic kidney disease; DM, Diabetes mellitus; HTN, Hypertension; HR, Hazards ratio; IQR, Interquartile range.*

**Supporting Table S12. Associations of peak/nadir liver biochemistries with death among patients with COVID-19, investigated with Cox proportional-hazards modelling**.

|  | Univariate analysis | | Multivariate analysis | |
| --- | --- | --- | --- | --- |
| Variables | Crude  HR (95% CIs) | p-value | Adjusted  HR (95% CIs) | p-value |
| Age (divided by 10 years) | 1.74 (1.53-1.97) | **<0.001** | **1.81 (1.56-2.09)** | **<0.001** |
| Gender (male) | 1.24 (0.91-1.71) | 0.18 | 1.23 (0.88-1.73) | 0.23 |
| Ethnicity (white) | 1.87 (1.25-2.8) | **0.002** | 1.07 (0.7-1.64) | 0.75 |
| Peak ALT (IU/L) | 1 (1-1) | 0.35 |  |  |
| Peak ALP (IU/L) | 1 (1-1) | 0.73 |  |  |
| Peak bilirubin (umol/L) | 1.01 (1-1.01) | 0.21 |  |  |
| (-Nadir albumin) ^†^ (g/L) | 1.07 (1.05-1.09) | **<0.001** | **1.07 (1.04-1.10)** | **<0.001** |
| Pre-existing Liver disease | 2.66 (1.44-4.9) | **0.002** | **2.42 (1.18-4.95)** | **0.016** |
| Pre-existing DM | 1.7 (1.16-2.48) | **0.006** | 1.32 (0.84-2.09) | 0.23 |
| Pre-existing HTN | 1.33 (0.96-1.84) | 0.085 | 0.72 (0.47-1.11) | 0.13 |
| Pre-existing CHD | 1.72 (1.11-2.65) | **0.015** | 1.06 (0.63-1.79) | 0.82 |
| Pre-existing CKD | 1.34 (0.83-2.17) | 0.23 | 0.74 (0.42-1.3) | 0.29 |
| Pre-existing Cancer | 1.71 (1.04-2.84) | **0.036** | 1 (0.57-1.74) | 1 |

^†^ To make it more interpretable, we transfer the nadir albumin values to negative values. NB. Analyses were performed on the whole COVID-19 group excluding one died patient with missing data on death date; variables (except for demographics and comorbidities) with p<0.1 in univariate analysis were included for multivariate analysis; in the multivariate analysis, HRs were fully adjusted for drugs use before baseline (including antiviral drugs, antibiotics, anticoagulants, acetaminophen, immunosuppressants, statins) to reduce confounding effects. *ALT, Alanine transaminase; ALP, Alkaline phosphatase; CHD, Coronary heart disease; CKD, Chronic kidney disease; DM, Diabetes mellitus; HTN, Hypertension; HR, Hazards ratio; IQR, Interquartile range.*

**Table S13. Predictive ability (AUC, Sensitivity, Specificity) of baseline liver biochemistry for mortality**

| **Variables** | **AUC** | **Sensitivity** | **Specificity** |
| --- | --- | --- | --- |
| Baseline albumin | 0.642 | 0.466 | 0.760 |
| Baseline ALP | 0.577 | 0.664 | 0.512 |
| Baseline BR | 0.554 | 0.555 | 0.564 |
| Baseline ALT | 0.546 | 0.712 | 0.428 |
| Baseline albumin + ALP + BR + ALT | 0.655 | 0.658 | 0.621 |

*Note: ALP, Alkaline phosphatase; ALT, Alanine transaminase; BR, bilirubin; ROC, receiver operating characteristic; AUC, the area under an ROC curve. Baseline albumin + ALP + BR + ALT indicates including all baseline liver biochemistry parameters in the ROC analysis.*

**Table S14. Predictive ability (AUC, Sensitivity, Specificity) of baseline liver biochemistry and other identified predictors of mortality**

| **Variables** | **AUC** | **Sensitivity** | **Specificity** |
| --- | --- | --- | --- |
| Age | 0.691 | 0.760 | 0.663 |
| Age + Liver disease | 0.711 | 0.795 | 0.610 |
| Age + Liver disease +  Baseline albumin | 0.752 | 0.801 | 0.607 |
| Age + Liver disease +  Baseline albumin + ALP + BR + ALT | 0.753 | 0.897 | 0.517 |

*Note: ALP, Alkaline phosphatase; ALT, Alanine transaminase; BR, bilirubin; ROC, receiver operating characteristic; AUC, the area under an ROC curve. Age (≥75 years or <75 years) and liver disease (yes/no) are used as binary variables, which were identified from multivariate Cox proportional-hazards models (Table 4). Baseline albumin + ALP + BR + ALT indicates including all baseline liver biochemistry parameters in the ROC analysis.*

**Supporting Table S15. Associations of baseline liver biochemistries with death among patients with COVID-19, investigated in subset/sensitivity analysis with Cox proportional-hazards models further adjusted for BMI**.

|  | Univariate analysis | | Multivariate analysis | |
| --- | --- | --- | --- | --- |
| Variables | Crude  HR (95% CIs) | p-value | Adjusted  HR (95% CIs) | p-value |
| Age (divided by 10 years) | 1.71 (1.44-2.03) | **<0.001** | **1.79 (1.47-2.18)** | **<0.001** |
| Gender (male) | 0.95 (0.64-1.42) | 0.82 | 1.20 (0.79-1.83) | 0.39 |
| Ethnicity (white) | 1.92 (1.11-3.33) | **0.02** | 1.18 (0.66-2.09) | 0.58 |
| BMI | 0.98 (0.95-1.01) | 0.22 | 1.01 (0.97-1.04) | 0.72 |
| Baseline ALT (IU/L) | 1 (0.99-1) | 0.34 |  |  |
| Baseline ALP (IU/L) | 1 (1-1) | 0.41 |  |  |
| Baseline bilirubin (umol/L) | 1.01 (0.99-1.02) | 0.42 |  |  |
| (- Baseline albumin) ^†^ (g/L) | 1.06 (1.02-1.09) | **0.003** | **1.04 (1.01-1.09)** | **0.049** |
| Pre-existing liver disease | 3.15 (1.53-6.51) | **0.002** | **2.98 (1.25-7.14)** | **0.014** |
| Pre-existing DM | 1.86 (1.18-2.94) | **0.008** | 1.38 (0.80-2.37) | 0.25 |
| Pre-existing HTN | 1.44 (0.96-2.16) | 0.077 | 0.86 (0.48-1.52) | 0.60 |
| Pre-existing CHD | 1.33 (0.74-2.39) | 0.33 | 0.76 (0.37-1.53) | 0.43 |
| Pre-existing CKD | 1.30 (0.74-2.30) | 0.36 | 0.81 (0.40-1.62) | 0.54 |
| Pre-existing Cancer | 1.99 (1.09-3.65) | **0.026** | 1.05 (0.51-2.17) | 0.89 |

^†^ To make it more interpretable, we transfer the baseline albumin values to negative values. *NB*. Analyses were performed on a subset of COVID-19 patients who had BMI and baseline liver biochemistry data (survived vs. died: 246 vs. 96); variables (except for demographics, comorbidities) with p<0.1 in univariate analysis were included for multivariate analysis; in the multivariate analysis, HRs were fully adjusted for drugs use before baseline (including antiviral drugs, antibiotics, anticoagulants, acetaminophen, immunosuppressants, statins) to reduce confounding effects. *ALT, Alanine transaminase; ALP, Alkaline phosphatase; CHD, Coronary heart disease; CKD, Chronic kidney disease; DM, Diabetes mellitus; HTN, Hypertension; HR, Hazards ratio; IQR, Interquartile range.*

**Table S16. Changes of liver biochemistries over the time assessed by linear mixed effects model for patients with COVID-19 within each subgroup (Died or Survived)**

| Liver biochemistries  in subgroup | Coefficient (β) | (95%CI) | p-value |
| --- | --- | --- | --- |
| **Albumin** |  |  |  |
| Died | -0.12 | (-0.14, -0.10) | <0.001 |
| Survived | 0.08 | (0.07, 0.08) | <0.001 |
| **ALP** |  |  |  |
| Died | 0.50 | (0.29, 0.70) | <0.001 |
| Survived | -0.07 | (-0.18, 0.03) | 0.164 |
| **ALT** |  |  |  |
| Died | 0.07 | (-0.55, 0.69) | 0.817 |
| Survived | -0.19 | (-0.28, -0.10) | <0.001 |
| **BR** |  |  |  |
| Died | 0.01 | (-0.02, 0.05) | 0.489 |
| Survived | -0.02 | (-0.03, -0.01) | <0.001 |

*Note: CI, confidence interval; ALP, Alkaline phosphatase; ALT, Alanine transaminase; BR, bilirubin.*

**Supporting Table S17. Detailed information regarding liver biochemistry recovery for each parameter in patients with and without COVID-19.**

| Test | Groups | Patients with ≥2 data points and deranged liver biochemistry | Patients had the liver biochemistry recovered  **n (%)** | Patients without liver biochemistry recovered  **n (%)** | p-value | Median (IQR) of latest liver biochemistry for patients did not had liver biochemistry recovered | p-value |
| --- | --- | --- | --- | --- | --- | --- | --- |
| ALT | COVID-19 group | 216 | 123 (56.9) | 93 (43.1) | p=0.32 | 71[55, 98] IU/L | p=0.47 |
|  | Non-COVID-19 group | 303 | 158 (52.1) | 145 (47.9) |  | 70[55, 112] IU/L |  |
| ALP | COVID-19 group | 194 | 86 (44.3) | 108 (55.7) | **p=0.21** | 176 [150, 240] IU/L | p=0.53 |
|  | Non-COVID-19 group | 366 | 141 (38.5) | 225 (61.5) |  | 168 [146, 229] IU/L |  |
| Bilirubin | COVID-19 group | 70 | 33 (47.1) | 37 (52.9) | **p=0.051** | 28[25,42] umol/L | p=0.13 |
|  | Non-COVID-19 group | 190 | 117 (61.6) | 73 (38.4) |  | 32[26,56] umol/L |  |
| Albumin | COVID-19 group | 438 | 151 (34.5) | 287 (65.5) | p=0.83 | 25[22,28] g/L | **p=0.005** |
|  | Non-COVID-19 group | 660 | 233 (35.3) | 427 (64.7) |  | 27[23,29] g/L |  |

**Supporting figures**

**Supporting Figure S1. Flowchart of patient selection for this study**. *ALT, Alanine transaminase; ALP, Alkaline phosphatase; ICU, intensive care unit.*

**
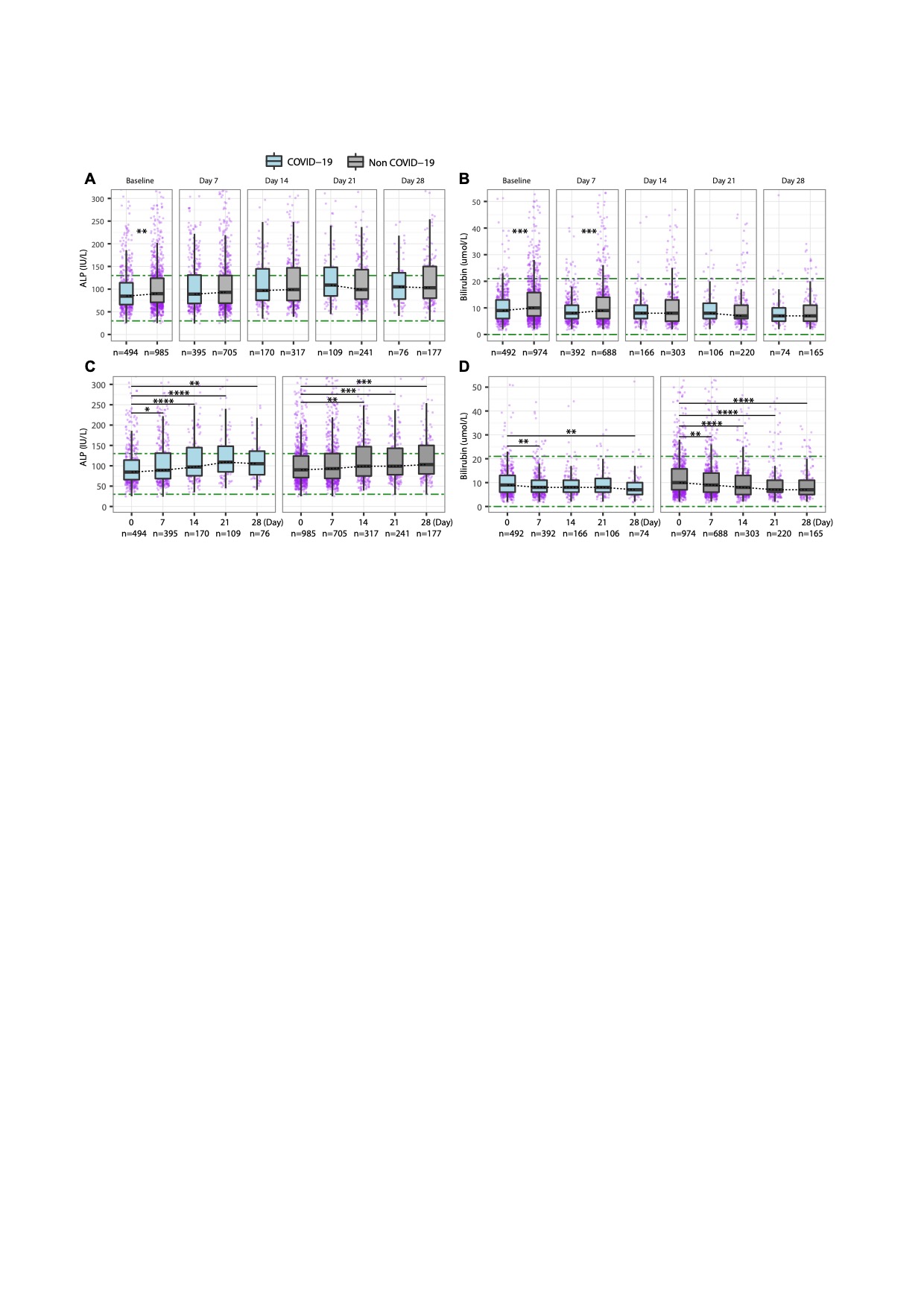
**

**Supporting Figure S2.** **Comparison of liver biochemistry (ALP, bilirubin) between COVID-19 group and non-COVID-19 group at each time point and the longitudinal changes of liver biochemistry (ALP, bilirubin) over time within each group**: (**A**) ALP comparison at baseline, 7, 14, 21, 28 days; (**B**) ALP changes over time; (**C**) Bilirubin comparison at baseline, 7, 14, 21, 28 days; (**D**) Bilirubin changes over time. *ALP, Alkaline phosphatase. Green dash-dotted lines indicate the lower limits of normal and the upper limits of normal. * p-value <0.05, ** p-value <0.01, *** p-value <0.001, **** p-value <0.0001*

**
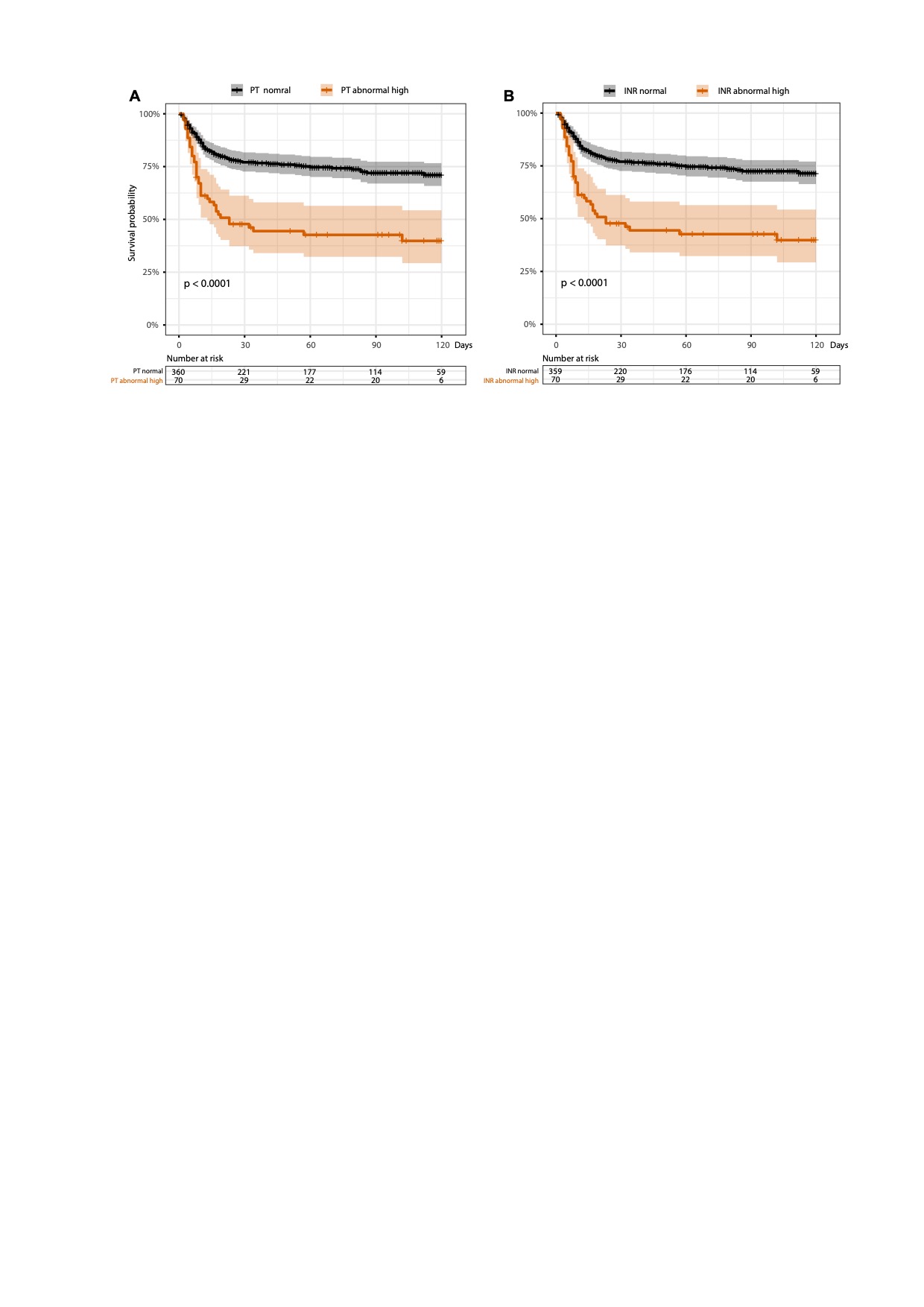
**

**Supporting Figure S3. K-M curves for comparisons of time from testing positive for SARS-CoV-2 to death for subgroups stratified by**: (**A**) Normal and abnormal high baseline PT; (**B**) normal and abnormal high baseline INR. *K-M, Kaplan-Meier; PT, Prothrombin time; INR, International normalised ratio. A subset of the COVID-19 group (n=430 vs. n=429) had data on PT and INR at baseline. p-values were based on the logrank test.*

**
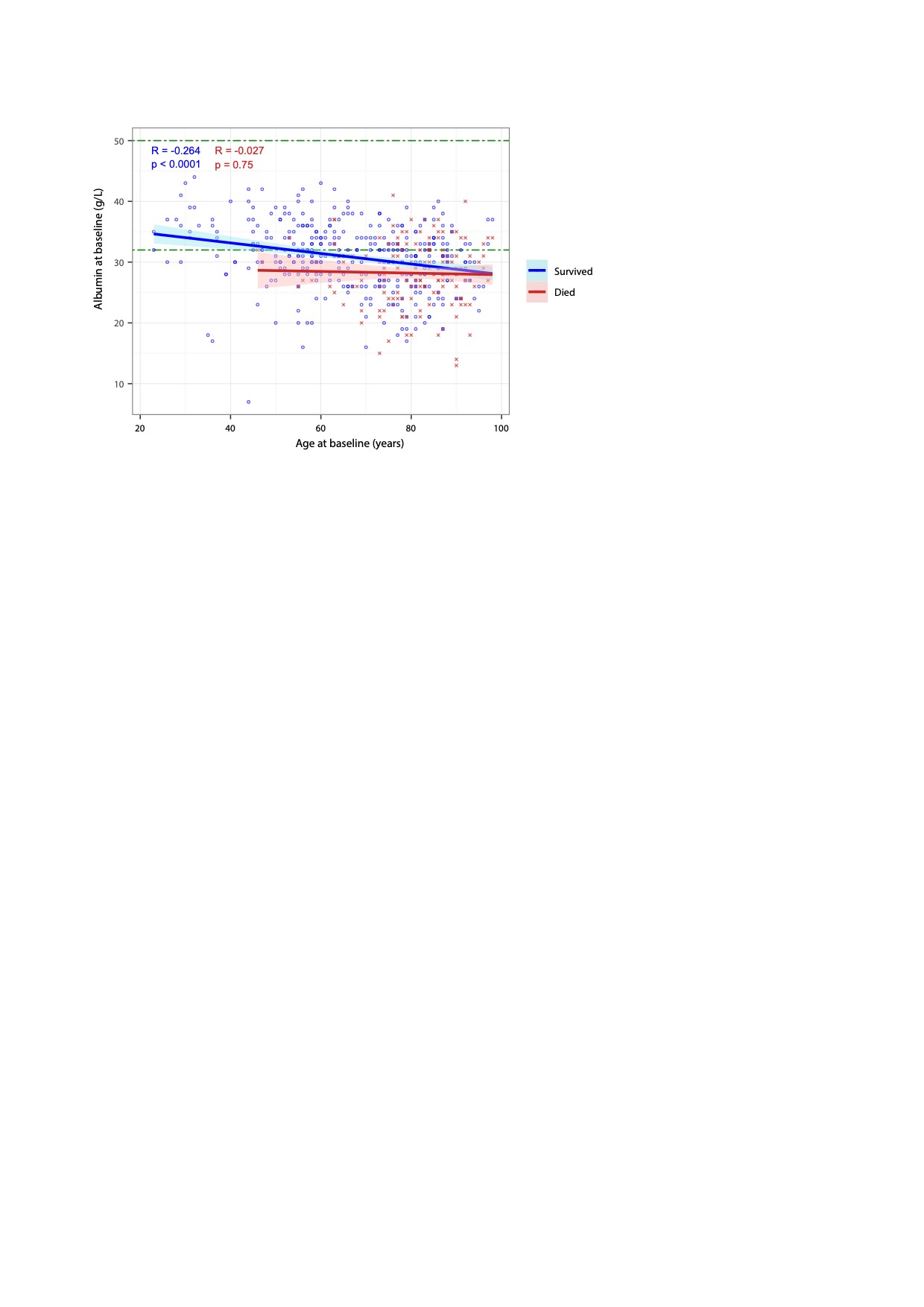
**

**Supporting Figure S4. Correlation of age with baseline albumin at the time of RT-PCR test in the COVID-19 group, stratified by survival status at end of follow-up**. *R represents Pearson’s correlation coefficient and p indicates linear regression significance.*

**
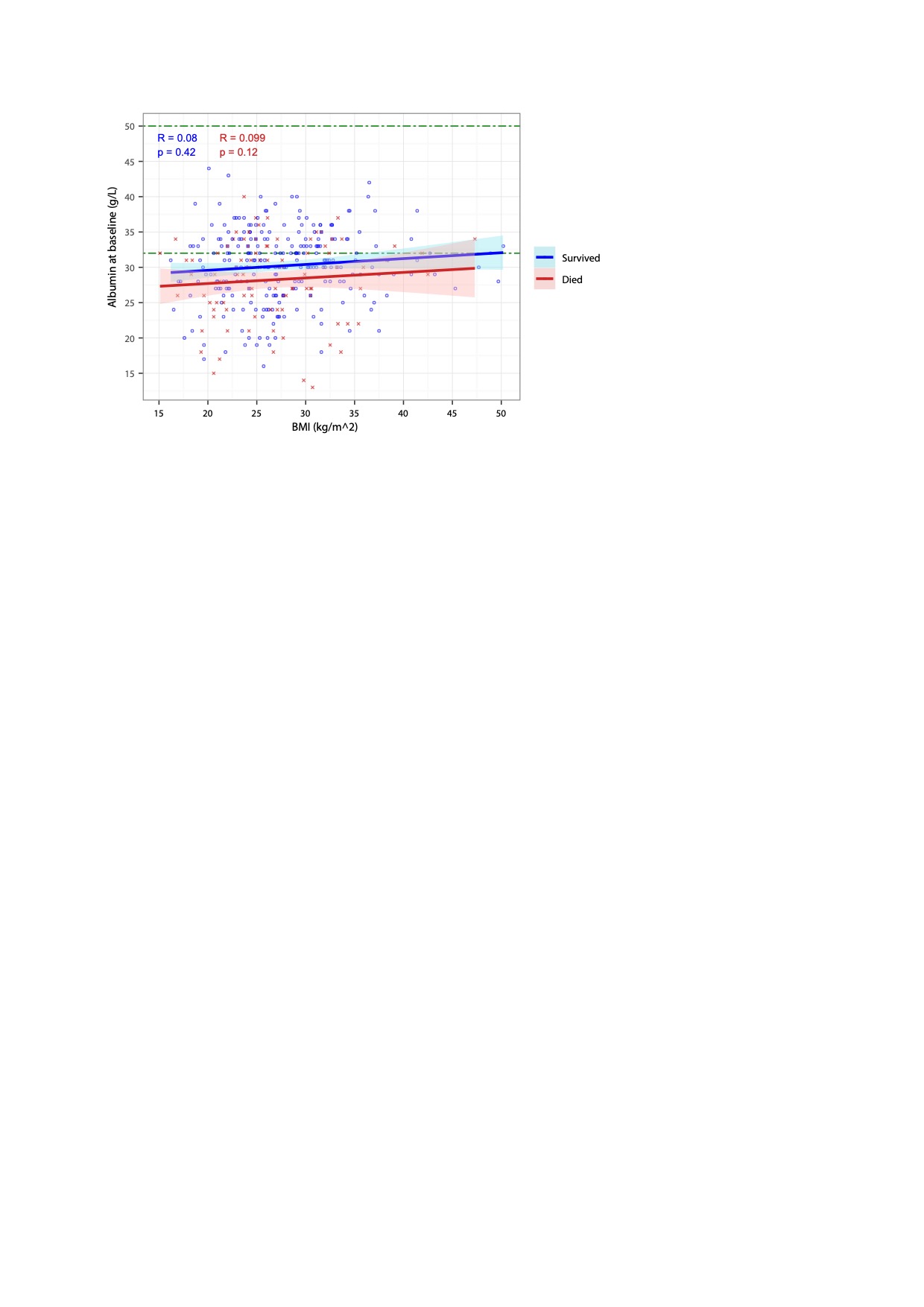
**

**Supporting Figure** **S5. Correlation of body mass index (BMI) with baseline albumin at the time of RT-PCR test in the COVID-19 group, stratified by survival status at end of follow-up**. *R represents Pearson’s correlation coefficient and p indicates linear regression significance.*


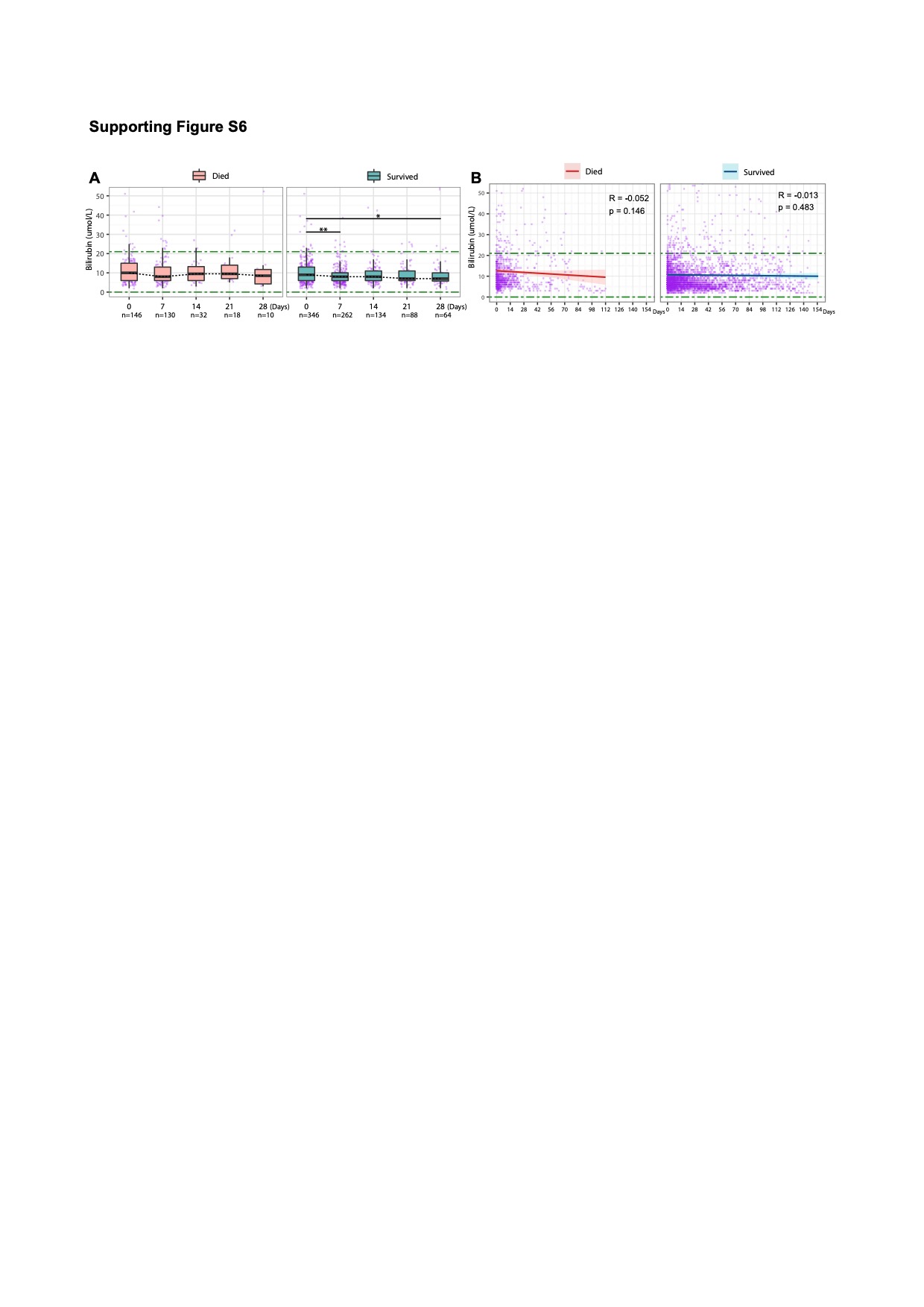


**Supporting Figure S6**. **Longitudinal changes of bilirubin over time of COVID-19 patients stratified by death during follow-up**. (**A**) Bilirubin at baseline at baseline, 7, 14, 21, 28 days; (**B**) Changing trend over time (112 vs. 155 days) of bilirubin by linear regression line fitting with 95% CI. *CI, Confidence interval. Green dash-dotted lines indicate the lower limits of normal and the upper limits of normal. * p-value <0.05, ** p-value <0.01. R represents Pearson’s correlation coefficient and p indicates linear regression significance.*
